# Zika virus encephalitis causes transient reduction of functional cortical connectivity

**DOI:** 10.1117/1.NPh.12.S1.S14603

**Published:** 2024-11-28

**Authors:** Shannon C. Agner, Lindsey M. Brier, Jeremy D. Hill, Ethan Y. Liu, Annie Bice, Rachel M. Rahn, Shengxuan Chen, Joseph P. Culver, Robyn S. Klein

**Affiliations:** a Washington University School of Medicine, Center for Neuroimmunology and Neuroinfectious Diseases, St. Louis, Missouri, United States; b Washington University School of Medicine, Department of Neurology, St. Louis, Missouri, United States; c Washington University School of Medicine, Department of Radiology, St. Louis, Missouri, United States; d Washington University School of Medicine, Department of Medicine, St. Louis, Missouri, United States; e Washington University School of Medicine, St. Louis, Missouri, United States; f Washington University School of Medicine, Departments of Physics, Biomedical Engineering, and Electrical and Systems Engineering, St. Louis, Missouri, United States; g Western University, Departments of Medicine, Microbiology & Immunology, Western Institute of Neuroscience, London, Ontario, Canada

**Keywords:** widefield optical imaging, flavivirus, microglia, astrocytes, neurons, mouse

## Abstract

**Significance:**

Determining the long-term cognitive impact of infections is clinically challenging. Using functional cortical connectivity, we demonstrate that interhemispheric cortical connectivity is decreased in individuals with acute Zika virus (ZIKV) encephalitis. This correlates with decreased presynaptic terminals in the somatosensory cortex. During recovery from ZIKV infection, presynaptic terminals recover, which is associated with recovered interhemispheric connectivity. This supports the contribution of synapses in the cortex to functional networks in the brain, which can be detected by widefield optical imaging. Although myeloid cell and astrocyte numbers are still increased during recovery, RNA transcription of multiple proinflammatory cytokines that increase during acute infection decreases to levels comparable to mock-infected mice during recovery. These findings also suggest that the immune response and cytokine-mediated neuroinflammation play significant roles in the integrity of brain networks during and after viral encephalitis.

**Aim:**

We hypothesized that widefield optical imaging would allow us to assess functional cortical network disruption by ZIKV, including hippocampal-cortical networks.

**Approach:**

We use widefield optical imaging to measure cortical functional connectivity (FC) in mice during acute infection with, and recovery from, intracranial infection with a mouse-adapted strain of ZIKV.

**Results:**

Acute ZIKV infection leads to high levels of myeloid cell activation, with loss of neurons and presynaptic termini in the cerebral cortex and associated loss of FC primarily within the somatosensory cortex. During recovery, neuron numbers, synapses, and FC recover to levels near those of healthy mice. However, hippocampal injury and impaired spatial cognition persist. The magnitude of activated myeloid cells during acute infection predicted both recovery of synapses and the degree of FC recovery after recovery from ZIKV infection.

**Conclusions:**

These findings suggest that a robust inflammatory response may contribute to the health of functional brain networks after recovery from infection.

## 1 Introduction

Zika virus (ZIKV) is a mosquito-borne member of the Flaviviridae family of viruses that re-emerged in the western hemisphere in 2015, most notably for teratogenic effects resulting from infections during pregnancy. However, ZIKV outbreaks throughout the world have continued to highlight neurologic consequences of ZIKV,^1^^,^^2^ indicating that, similar to other vector-borne Flaviviruses,^3^ ZIKV can impact the adult central nervous system (CNS).^4^^–^^9^ There is growing evidence that long-term cognitive sequelae occur after systemic ZIKV infection, even in the absence of severe illness or encephalitis.^10^^,^^11^ Further, these results do not seem to be restricted to older patients, as the neurocognitive decline has been also observed in adolescents^12^ and young adults.^13^ Although the extent of ZIKV effects in the hippocampus has been previously studied in murine models by our laboratory and others,^14^^–^^18^ little is known about its acute effects on the cerebral cortex, including the health of brain networks, and how they might contribute to neurocognitive changes after recovery from infection.

One method to characterize brain networks is an examination of functional connectivity (FC), which is accomplished via the evaluation of regional correlations between intrinsic neural activity that are necessary for cerebral homeostasis and cognition.^19^^–^^21^ The functional magnetic resonance imaging (fMRI) community has made great strides in mapping the connections necessary for multiple brain processes using seed-based analysis to calculate the temporal synchrony between any two brain regions at rest (i.e., “resting state FC”, rs-FC).^22^^,^^23^ fMRI of encephalitic patients offers unique challenges to the neuroimaging community because altered mental status, which is common in encephalitis patients, results in intolerance for lengthy scans necessary for functional analysis.^24^ Thus, although structural MRI is used as a diagnostic tool for encephalitis, functional neuroimaging studies have not been previously done.

In contrast to fMRI, optical imaging can be performed with fewer restrictions, reduced cost, and greater availability than fMRI. In animal models, an optical approach [widefield optical imaging (WFOI)] has elucidated cortical FC networks in the mouse.^25^^,^^26^ This method also has added advantages of imaging study subjects while awake and with head fixation, increasing data fidelity and eliminating the effects of sedation and motion artifacts on functional networks. Previous WFOI studies in mice have shown sensitivity to disease and have elucidated local perturbations associated with glioma growth,^27^ stroke,^28^ and Alzheimer’s disease (AD).^29^ This modality, however, has not been extended to animal models of neurotropic and emerging Flavivirus infections that induce long-term cognitive sequelae, which may lead to dementia.^30^

In this study, we evaluated cortical FC in an adult murine model of ZIKV infection. Eight-week-old Thy1-GCAMP6f mice were intracranially (i.c.) infected with a mouse-adapted Dakar strain (ZIKV-Dak-MA), which exhibits significant tropism for the mouse brain,^31^ particularly in neurons of the hippocampus and cerebral cortex.^31^ FC was compared between ZIKV-Dak-MA- and mock-infected mice during acute infection and after viral clearance and recovery. Deficits in interhemispheric FC were observed in the somatosensory cortex during acute infection and improved during recovery, despite continued memory deficits observed using behavioral testing. These changes correlated with alterations in the presynaptic marker density, synapsin, which also recovered after the resolution of the infection. Although myeloid cell activation did not appear to correlate with region-specific FC during acute infection, the degree of persistent myeloid cell activation during recovery did correlate with the degree of recovery of FC. Conversely, astrocyte activation was anti-correlated with the degree of recovery of FC. Although cortical brain networks appeared to be intact after recovery, severe hippocampal injury, with associated impairment in spatial learning and memory persists after recovery from ZIKV-Dak-MA infection. These data indicate that cortical networks may recover in the setting of subcortical injury, but this does not contribute to the recovery of hippocampal-based learning tasks. Importantly, cytokine activation during acute infection and deactivation during recovery from infection contributes to functional brain networks.

**Table 1:** Description of mice used for each experiment.

Experiment	Male mice	Female mice	Total number of mice
Viral titers at 3, 7, and 15 days post-infection	27	0	27
Survival, weights, encephalitis scores, and behavior	68	0	68
Widefield optical imaging, and histochemical staining	21	24	45
Imaging control study: effect of needle insertion	0	8	8

## 2 Materials and Methods

### 2.1 Animals

A total of 148 mice were used in this study (Table 1). For imaging studies, a total of 45 two-month-old mice (mock, N=25, ZIKV, N=26) consisting of 24 female and 21 male mice in total (N=12 female, N=10 male mock and N=12 female, and N=11 male ZIKV mice) *Thy1*-GCaMP6f mice were used (Jackson Laboratories Strain: C57BL/6J-*Tg(Thy1-GCaMP6f)GP5*.*5Dkim*; stock: 024276). These mice express the protein GCaMP6f in excitatory neurons, primarily in cortical layers ii, iii, v, and vi.^26^ All studies were approved by the Washington University School of Medicine Animals Studies Committee and follow the guidelines of the National Institutes of Health’s Guide for the Care and Use of Laboratory Animals.

To evaluate for effects of intracranial needle insertion, mock mice are compared with mice injected peripherally with PBS 72 h prior (N=8, all females, 3 to 7 months old) (Fig. S3 in the Supplementary Material). These mice underwent typical surgical preparations as described below, with additional bilateral EEG implantation (not used in the present study).

### 2.2 Zika Virus Infection

**Fig. 1:**
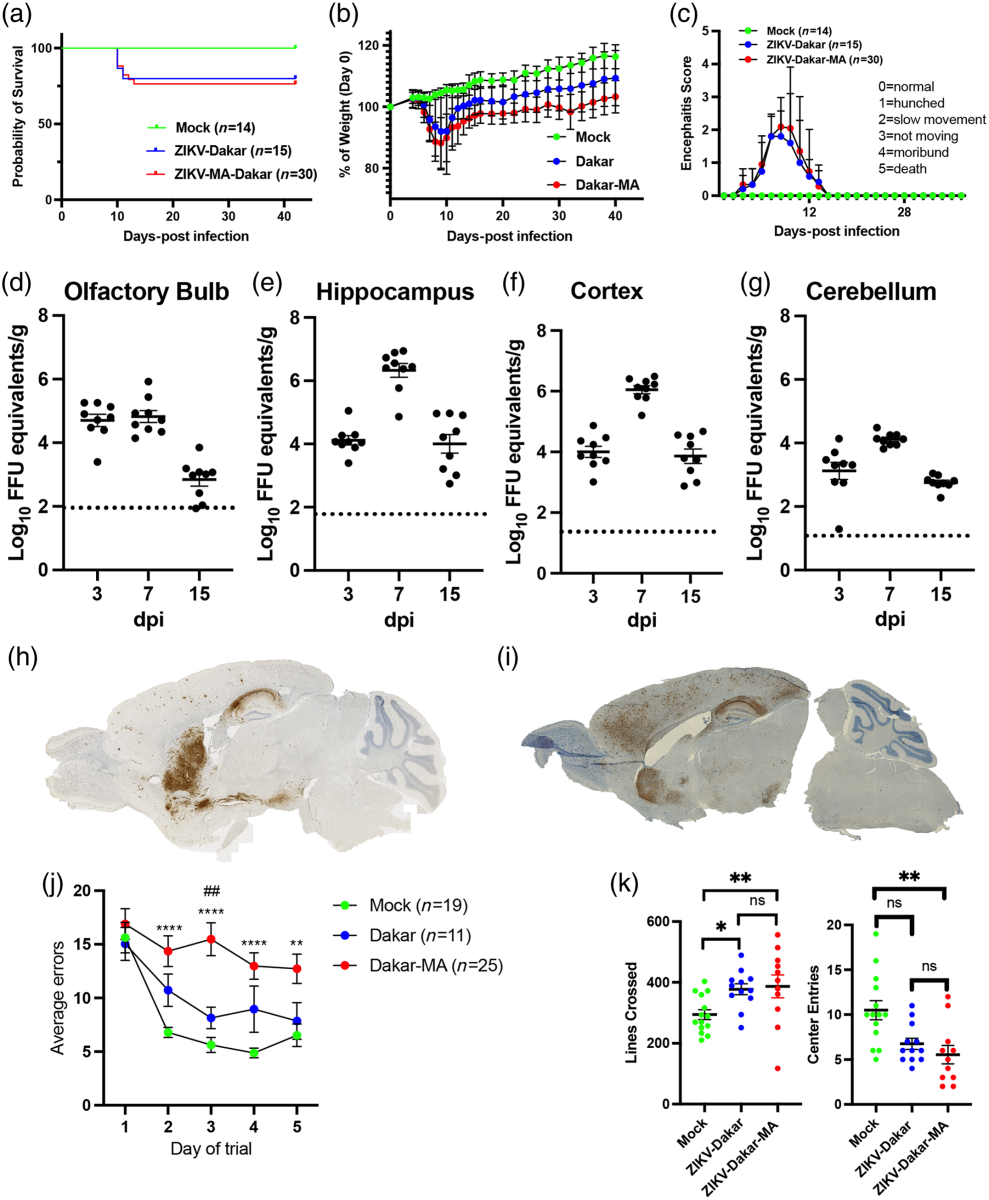
Mouse-adapted strain of ZIKV-Dakar has similar viral kinetics to the parent strain but more widespread cortical infection and more severe cognitive effects. (a) Survival curves and (b), (c) weights and encephalitis scores comparing MA and parent Dakar strain [mock, N=14; ZIKV-Dakar, N=15; and ZIKV Dakar-MA, N=30 for panels (a)–(c)]. (d)–(g) Viral titer levels at 3, 7, and 15 days after intracranial infection. N=9 for each timepoint. The dotted lines represent the limit of sensitivity of the assay. *In situ* hybridization on sagittal mouse brain sections of (h) parent and (i) mouse-adapted Zika strains. Behavioral testing data showing (j) Barnes maze and (k) open field data comparing mock, parent strain Dakar, and MA Dakar-infected mice at 42 days post-infection. For panel (j), a two-way ANOVA with correction for multiple comparisons was performed. Data for each treatment group (mock- vs. Dakar vs. MA-Dakar) were compared on each trial date. The number of errors for MA-Dakar mice was significantly different from the mock-infected mice on days 2–5 of testing. In addition, the number of errors for the MA-Dakar mice was significantly different (P=0.0016) from Dakar mice on day 3 of testing but not any other days of testing. Quantification of open field testing (k) by lines crossed (k, left) and center entries (k, right). The black horizontal bar represents the mean. Data were analyzed by two-way ANOVA and corrected for multiple comparisons. *P<0.05, **P<0.01.

The ZIKV mouse-adapted (MA) Dakar strain utilized for intracranial infections was obtained from M. Diamond at Washington University in St Louis. The MA Dakar strain of ZIKV was obtained by passage of the original Dakar strain of ZIKV through a *Rag* -/- mice,^31^ resulting in a strain of ZIKV that was found to replicate more efficiently in the mouse brain than the parent strain. For the evaluation of viral kinetics, a total of 27 C57BL/6J male mice were intracranially infected with ZIKV MA Dakar, and the brain tissue was harvested during early infection 3 days post-infection (dpi), peak infection (7 dpi), and early infection recovery (15 dpi). Timepoints were chosen based on predicted timelines from previous publications of the original Dakar strain of ZIKV^14^ as well as younger mice intracranially infected with ZIKV MA Dakar.^31^ Mice were deeply anesthetized using 4% isoflurane for induction and 1% to 2% isoflurane for maintenance via nose cone. During maintenance anesthesia, mice were intracranially administered $1\times 10^{4}$ plaque-forming units (p.f.u.) of ZIKV MA Dakar. Viruses were diluted in 10 μl of 0.5% fetal bovine serum in Hank’s balanced salt solution (HBSS) and injected into the midline of the brain with a guided 29-gauge needle. The target location of Bregma is approximated at 2/3 distance between an imaginary line between the eyes and an intra-aural line. Depth of injection is consistent using a custom-designed spacer guide (11 mm) through which injection occurs with a 13 mm length needle. Thus, the depth of injection is consistently 2 mm. The location of injection was confirmed at the time of surgical windowing, and no mice had an off-target injection by more than +/− 1 mm from the target location in either the anteroposterior axis or mediolateral axis.^32^^,^^33^ This technique for injection was chosen over stereotaxic injection due to the minimized duration of anesthesia, which can affect synaptic density and the health of neurons.^34^ Mock-infected mice were intracranially injected with 10  μl of 0.5% fetal bovine serum in HBSS into the midline of the brain with a guided 29-gauge needle. To evaluate survival and confirm expected infection and recovery, mice were weighed and encephalitis scores were evaluated for a total of 68 mice in the behavioral testing experiments [Figs. 1(a)–1(c)]. The encephalitis score used has been previously published in models of infectious encephalitis.^14^ The scoring system is as follows: 0 = normal; 1 = hunched; 2 = slow movement; 3 = not moving; 4 = moribund; and 5 = dead.

### 2.3 Surgical Windowing

Immediately after the intracranial infection was completed, a cranial imaging window was placed. A plexiglass optically transparent window was implanted with translucent dental cement (C&B-Metabond, Parkell Inc., Edgewood, New York) following a midline incision and clearing of the skin and periosteal membranes. The window covered the majority of the dorsal cortical surface, provided an anchor for head fixation, and allowed for chronic, repeatable imaging.^35^

### 2.4 Immunohistochemistry

After imaging at either 7 dpi or 42 dpi on the same day, mice were anesthetized and perfused with ice-cold Dulbecco’s PBS (Gibco) followed by ice-cold 4% paraformaldehyde (PFA). Brains were post-fixed overnight in 4% PFA followed by cryoprotection in 30% sucrose (three exchanges for 24 h), then frozen in OCT compound (Fisher Scientific, Massachusetts, United States). Coronal tissue sections (10  μm) were washed with PBS and permeabilized with 0.1% to 0.3% Triton X-100 (Sigma-Aldrich, St. Louis, United States). Nonspecific antibodies were blocked with 5% normal goat serum (Sigma-Aldrich) at room temperature for 1 h. To reduce endogenous mouse antibody staining when detecting ZIKV, a Mouse on Mouse kit (MOM basic kit, Vector) was used as per the manufacturer’s instructions. Slides were then incubated in primary antibody (described below) or isotype-matched IgG overnight at 4°C. Following washes in PBS, slides were then incubated in secondary antibodies at room temperature for 1 h, and nuclei were counterstained with 4,6-diamidino-2-phenylindole (DAPI; Invitrogen, Waltham, United States). Coverslips were applied with ProLong Gold Antifade Mountant (Thermo Fisher, Waltham, United States). Immunofluorescent images were acquired using a Zeiss LSM 880 confocal laser scanning microscope and processed using software from Zeiss. Images at 20× were acquired of layers II to VI of the somatosensory cortex. Images at 63× were acquired of layers II to IV. Immunofluorescent signals were quantified using the software ImageJ and using in-house written scripts using Matlab (Natick, MA, United States).

### 2.5 RNAscope *In situ* Hybridization

Tissue was prepared similarly to that used for immunohistochemistry. RNAscope 2.5 HD Assay-Brown was performed as per the manufacturer’s instructions. Probes against ZIKA mRNA (ACD) were used. Viral tropism was analyzed using a ZEISS Axio Imager Z2 fluorescence microscope.

### 2.6 Quantitative Reverses Transcription PCR

Forty-micron thick fixed tissue sections that were previously suspended in cryoprotectant were washed in cold PBS and then dissected into cortical and hippocampal sections. The tissue was then suspended in proteinase K solution. RNA was extracted from the tissue using the Qiagen RNeasy FFPE Kit (Cat no. 73504) per the manufacturer’s instructions. Conversion to cDNA was performed using a high capacity reverse transcriptase cDNA kit (Thermo Fisher, # 4368813). Viral RNA was quantified using the IDT Prime Time gene expression master mix (#1055772). Cytokine RNA was quantified using Power SYBR green master 455 mix (Thermo Fisher, #4367659) and custom IDT primers. All qPCRs were performed in 384 well plates. Unless otherwise specified, all data are reported as $2^{-\Delta \Delta \mathrm{CT}}$ relative to glyceraldehyde-3-phosphate dehydrogenase (GAPDH), which is a commonly used control for RNA expression due to the role of GAPDH as a consistently and ubiquitously expressed (i.e., “housekeeping”) reference gene in adult mice.^36^ Primers used have all been previously published and are as follows:

C1q-F (TCTGCACTGTACCCGGCTA), C1q-R (CCCTGGTAAATGTGACCCTTTT);^37^ Il1b-F (ACCTGTCCTGTGTAATGAAAGACG), and Il1b-R (TGGGTATTGCTTGGGATCCA);^37^ Tnfa-F (TGTGCTCAGAGCTTTCAACAA) and TNFa-R (CTTGATGGTGGTGCATGAGA);^37^ Sparc-F (TGGGAGAATTTGAGGACGGTG) and Sparc-R (GAGTCGAAGGTCTTGTTGTCAT);^38^ Mertk-F (CTGCTTCTGCGGGTTTGTTC) and Mertk-R (GGCTTTGCAAGGTA AGCTCG);^39^ Megf10-F (CCAGCCAACAGGAATGTCTAT) and Megf10-R (ACTGGCAGCAGGTCATAATG);^40^ IFNg-F (AACGCTACACACTGCATCTTGG) and IFNg-R (GCCGTGGCAGTAACAGCC);^41^ IFNb-F (CTGGAGCAGCTGAATGGAAAG) and IFNb-R (CTTCTCCGTCATCTCCATAGGG);^41^ IL6-F (CTCCGCAAGAGACTTCCAG) and IL6-R (GGTCTGTTGTGGGTGGTATC);^42^ IFNalpha-F (CTTCCACAGGATCACTGTGTACCT) and IFNalpha-F (TTCTGCTCTGACCACCTCCC);^43^ and GAPDH-F (GGCAAATTCAACGGCACAGT) and GAPDH-R (AGATGGTGATGGGCTTCCC).^44^

### 2.7 Widefield Optical Imaging

Mice were head-fixed in a stereotaxic frame and body secured in a black felt pouch for imaging. Sequentially firing LEDs (Mightex Systems, Pleasanton, California, United States) passed through a series of dichroic lenses (Semrock, Rochester, New York) into a liquid light guide (Mightex Systems) that terminated in a 75 mm f/1.8 lens (Navitar, Rochester, New York) to focus the light onto the dorsal cortical surface. LEDs consisted of 470 nm (GCaMP6f excitation), 530, 590, and 625 nm light. An sCMOS camera (Zyla 5.5, Andor Technologies, Belfast, Northern Ireland, United Kingdom) coupled with an 85 mm f/1.4 camera lens (Rokinon, New York, New York) was used to capture fluorescence/reflectance produced at 16.8 Hz per wavelength of LED. A 515 nm longpass filter (Semrock) was used to discard GCaMP6f excitation light. Cross polarization (Adorama, New York, New York) between the illumination lens and collection lens discarded artifacts due to specular reflection. The field-of-view (FOV) recorded covered the majority of the convexity of the cerebral cortex ($∼1.1\;{\mathrm{cm}}^{2}$), extending from the olfactory bulb to the superior colliculus. All imaging data were acquired as 5-min runs and binned in $156\times 156\;{\text{pixel}}^{2}$ images at ∼100  μm per pixel. All 45 mice were imaged at 7 dpi, whereas a smaller cohort (mock, N=11, ZIKV, N=8) was imaged post-recovery at 42 dpi.

The projection of the three-dimensional, convex mouse cortex to a flat-field projection is considered in two ways in the analysis of this dataset. The first is that the illumination is not even across the mouse head. This is accounted for by normalizing the data to the average light intensity within each pixel, which normalizes any illumination heterogeneities. The second is that given the mouse head is not flat, and has some convexity to it, WFOI provides essentially a projection from a three-dimensional shape. However, the curvature is only on the order of 1 mm z-displacement across the field of view.^45^ Further, to account for this, anatomical locations, seed placement, and functional labeling are also based on a projection from the Paxinos atlas to our WFOI field-of-view.^25^

Both infraslow and delta frequency filters were used in our analyses. fMRI studies use an infraslow filter to capture the majority of the slow hemodynamic response, and thus, processing our hemoglobin data within this band allows us the ability to compare our results with existing fMRI literature. However, this slower frequency band is more prone to high amplitude and low-frequency movement artifacts compared with higher frequency bands (e.g., delta). We complement this infraslow hemoglobin data with the calcium data filtered through delta band frequencies, thereby providing a more robust signal-to-movement noise result. It is worth noting that respiratory and cardiac physiologic signals could be captured within the 0.4 to 4.0 Hz frequency range. We would expect any signal coming from the brain periphery (e.g., cardiac, respiratory) to affect our recordings globally. To this end, we use global signal regression (GSR) to filter out global changes in brain dynamics. As evidenced in Fig. S4 in the Supplementary Material, these pre-processing steps were successful in yielding low global fluctuations. One critique of GSR is the induction of spurious anti-correlations. Using simulation analysis, Murphy et al.^46^ demonstrated the shift of correlation values from a left-skewed distribution to a normal distribution centered around 0 when performing GSR on Pearson correlation data. For consistency, we processed all imaging data points through the same pre-processing steps, so it is possible that GSR has mathematically induced anti-correlations seen in this work and elsewhere. However, we restrict our statistical analysis to compare changes induced by ZIKV with controls and refrain from commenting on brain networks in a single sample at a single timepoint.

### 2.8 Behavioral Testing

Animals underwent open-field and Barnes maze testing as previously described.^14^ Briefly, mice were given 3 min to explore the Barnes maze platform and to find the target hole. If a mouse did not find the target hole within the test period, it was gently guided into the hole. At the end of a trial, the mice remained in the target hole for 1 min before being placed back into their home cage between trials. Each mouse received two trials per day over the course of five consecutive days. The number of errors (or number of nose pokes over non-target holes) was recorded. Before Barnes maze testing, animals performed the open field test as a measure of exploratory behavior. Each mouse was given 5 min to explore an empty arena before being placed back into its home cage. Both the Barnes maze platform and the open-field arena were decontaminated with 70% ethanol between trials and/or mice. All trials were video recorded using a camera (Canon Powershot SD1100IS) and scored by experimenters blinded to the treatment conditions.

### 2.9 Statistical Analysis for Virologic Studies, Behavioral Studies, and Histochemical Assays

Unpaired Student’s t test or one-way or two-way analysis of variance (ANOVA) with appropriate post-test to correct for multiple comparisons, in which the means of all groups were compared pairwise, were performed as indicated in the figure legends. Prism 10.0 (GraphPad Software) was used for generating graphs and statistical analyses. P values of ≤0.05 were considered significant, and Cohen’s effect size, d, is reported with results (Table S1 in the Supplementary Material). For histochemical analysis, sample sizes were chosen based on a combination of power calculations from previously published data and previously published sample sizes. Using data from quantitation of similar stains of the hippocampus in Garber et al.,^14^ for a probability of type I error = 0.05 and power =90%, a sample size of 2 for each of mock and infected mice is needed (95% CI = [60.16;70.98]). However, given that the cerebral cortex had not been evaluated before, a sample size of five per group was chosen to account for the potential of larger variance in the cortex as well as to align with previously published sample sizes.^14^^,^^44^ No a priori power calculations were done for virologic studies or behavioral testing. Rather, the sample size was chosen based on prior studies where available.^14^^,^^44^ For all experiments, animals were randomly assigned to mock or MA-ZIKV-Dak infection. Investigators were blinded to group allocation during data collection and analyses. A Pearson correlation coefficient was computed to assess the linear relationship between cellular content and interhemispheric connectivity. Prism 10 was used for Pearson correlation analysis.

### 2.10 Image Data Processing and Analysis

Image processing followed methods previously described^25^^,^^47^ and briefly summarized here. Images were spatially downsampled to $78\times 78\;{\text{pixel}}^{2}$, and a frame of ambient baseline light levels was subtracted from the time series data. Data were temporally downsampled by a factor of 2 and then spatially and temporally detrended. Data were affine-transformed to common Paxinos atlas space using lambda (where the superior colliculus and cerebrum intersect at midline) and the anterior suture (where the olfactory bulb and cerebrum intersect at midline) as anatomical landmarks. Pixel-wise time traces were then mean normalized and regressed from the time series data as described in Ref. 48. Frames corresponding to reflection data produced by the 530, 590, and 625 nm light were used to solve the modified Beer-Lambert law to yield fluctuations in oxygenated and deoxygenated hemoglobin. Frames corresponding to fluorescence data were corrected by approximating hemoglobin absorption of the excitation and emission light. All data were spatially smoothed with a 5×5 Gaussian filter (sigma = 1.2). To track motion noise, we used a measure from the fMRI and human optical fields, where we calculated the temporal derivative of each pixel and the sum of the square of that signal to generate a global variance in the temporal derivative signature (GVTD).^49^ This measure increases in the presence of motion. We find no significant differences between the Zika group and the control group (Fig. S4 in the Supplementary Material).

For FC analysis, we calculated the Pearson correlation coefficient between the time traces from two different regions

$$\rho_{x,y}=\frac{\mathrm{cov}(x,y)}{\sigma_{x}\sigma_{y}},$$ (1)

where $\rho_{x,y}$ is the Pearson correlation coefficient between time traces x and y, cov(x,y) is the covariance between time traces x and y, and $\sigma_{x}$ and $\sigma_{y}$ are the standard deviations within time trace x and time trace y, respectively. Bilateral FC maps represent Pearson correlation coefficients calculated between pixel-wise time traces on the left hemisphere and their corresponding symmetrical right hemisphere pixel-wise time trace. Power spectral analysis of the GCaMP6f data was performed on 10-s segments by applying a Hann window and an FFT (squared to obtain power).

#### 2.10.1 Imaging data statistics

A challenge with analyzing the statistical significance in functional imaging is managing the multiple comparisons problem. Here, we used a cluster size-based method that leverages the spatial connection between pixels and credits large clusters as having more statistical significance than small clusters with the same peak t value. More specifically, we used a cluster size-based thresholding method to analyze FC maps and to ensure the family-wise error rate of any threshold map did not exceed p=0.01.^48^

## 3 Results

### 3.1 Infection of Thy1-GCAMP6f Mice with ZIKV-Dak-MA Leads to Destruction of Hippocampus with Loss of Spatial Learning Ability

Previous data demonstrated that ZIKV targets the hippocampi of adult mice after i.c. infection with an African strain of ZIKV from Dakar, Senegal (ZIKV-Dak).^14^ Recent data demonstrated more severe CNS infection using a mouse-adapted strain of ZIKV-Dak (ZIKV-Dak-MA).^31^ Prior studies using WFOI have demonstrated the ability to detect disrupted functional networks in the setting of subcortical injury and given the extent of ZIKV-Dak-MA infection in the hippocampus and throughout the cortex, we hypothesized that WFOI would allow us to assess functional cortical network disruption by ZIKV, including hippocampal-cortical networks.

To determine the extent of infection and cognitive correlates of this more severe ZIKV brain infection, 8-week-old C57Bl/6J mice were intracranially (i.c.) inoculated with either 5% fetal bovine serum in phosphate-buffered saline (i.e., mock), ZIKV-Dak, or ZIKV-Dak-MA. Survival, weight loss, and encephalitis scores of mice infected with ZIKV-Dak-MA were similar to those seen in mice infected with ZIKV-Dak [Figs. 1(a)–1(c)]. We also found that 8-week-old C57Bl/6J mice had similar levels of ZIKV-Dak-MA viral replication in the olfactory bulb, hippocampus, cortex, and cerebellum [Figs. 1(d)–1(g)] compared with 4- to 5-week-old C57Bl/6J mice, published previously.^31^ Using *in situ* hybridization to assess viral RNA, we found that ZIKV-Dak-MA exhibited tropism for the hippocampus, similar to ZIK-Dak,^14^ but also infected the cerebral cortex at 7 days post-infection (dpi) [Figs. 1(h), 1(i)]. Evaluation of spatial learning via the 5-day Barnes maze spatial learning paradigm (as described previously^44^) at 42 dpi revealed more significant numbers of errors in ZIKV-Dak-MA- versus mock-infected mice and had significantly more errors on day 3 of the trial compared with the ZIKV-Dak-recovered mice [Fig. 1(j)]. ZIKV-Dak-recovered mice were slower to learn the maze than mock-infected animals but were eventually able to learn it. By contrast, ZIKV-Dak-MA-recovered animals were completely unable to learn the spatial learning paradigm. No significant differences in mobility were found, which is tested in the open field test by quantifying the number of lines crossed in the demarcated box [Fig. 1(k)]. In addition, there were no significant differences between ZIKV-Dak- and ZIKV-Dak-MA-infected mice in center entries in the open field test, which measures anxiety by evaluating the proportion of time the mice spend in the center of the box compared with time spent in the periphery^44^ [Fig. 1(k)]. These data suggest that ZIKV-Dak-MA induces a complete loss of spatial learning ability after recovery from acute infection.

### 3.2 Neuronal Injury and Inflammation in the Hippocampus During Acute ZIKV Infection Persist After Recovery

To understand the cellular correlates of ZIKV-Dak-MA infection and the significant spatial learning deficits noted after recovery from ZIKV-Dak-MA infection, we first assessed the hippocampus at 7 dpi. Quantitative immunohistochemical (IHC) detection of neurons (NeuN), activated myeloid cells (Iba1-positive), and activated astrocytes (GFAP-positive) at 7 dpi revealed decreases in numbers of NeuN+ cells, and increased GFAP+ and IBA1+ cells [Figs. S1(A)–S1(E) in the Supplementary Material], indicating injury to the hippocampus and inflammation in the region of the hippocampus, even during acute infection. To determine if this injury persisted or recovered after viral clearance and recovery from infection, quantitative immunohistochemical (IHC) detection of neurons (NeuN), activated myeloid cells (Iba1-positive) and activated astrocytes (GFAP-positive) at 42 dpi revealed severe injury in the cornu ammonis, region 3 (CA3) of the hippocampus with persistent inflammatory response, including increased GFAP expression, increased activated myeloid cells, and decreased numbers of neurons when compared with mock-infected animals [Figs. S1(F) and S1(G) in the Supplementary Material]. This persistent injury to the hippocampus was much more severe than previously reported for ZIKV-Dak^14^ and suggests more extensive destruction of the hippocampus results in the inability of mice infected with ZIKV-Dak-MA to learn the Barnes maze despite virologic control [Fig. 1(j)].

### 3.3 Acute ZIKV Infection Causes Decreased Cortical Connectivity in the Somatosensory Cortex

**Fig. 2:**
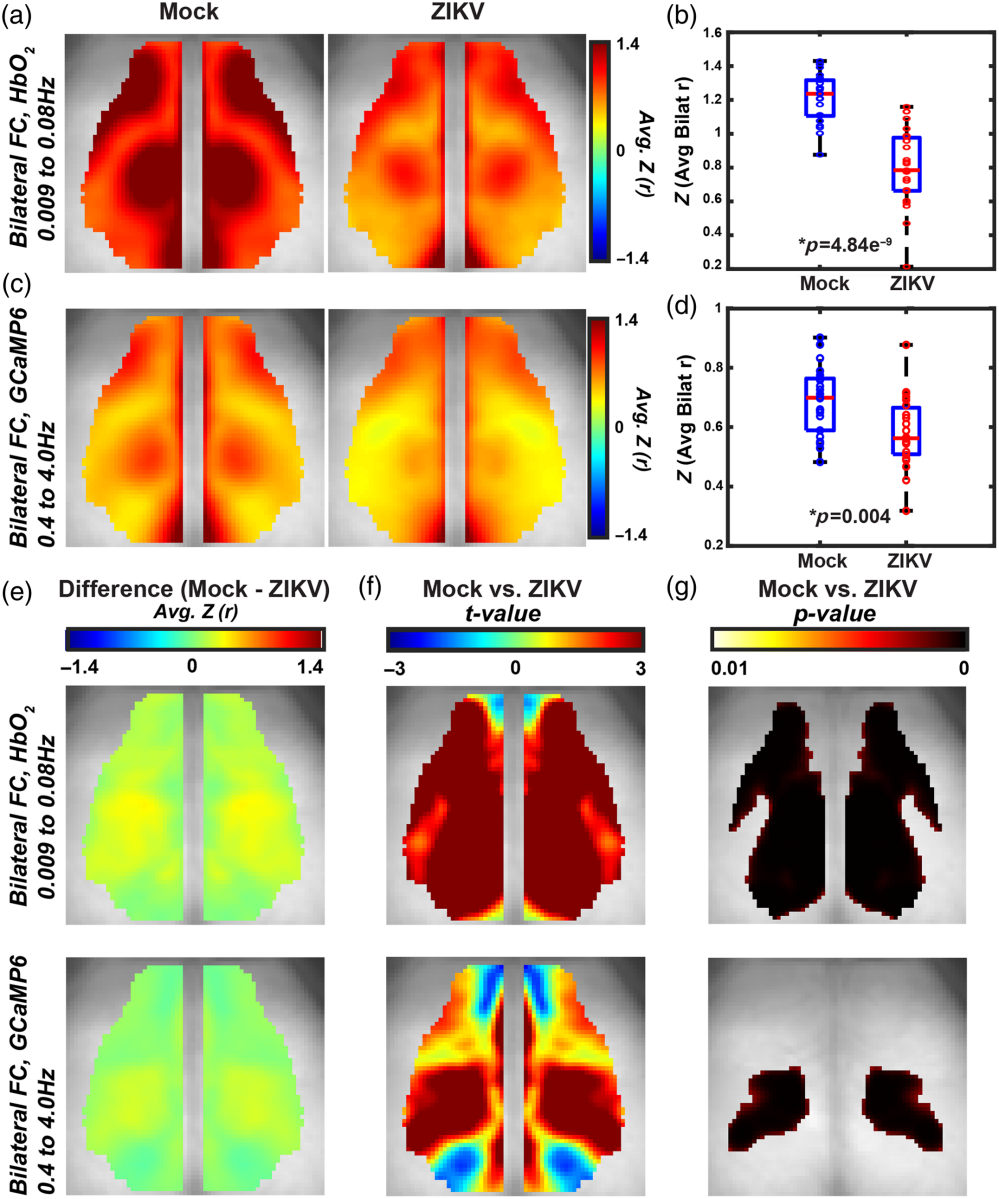
ZIKV infection reduces delta calcium and infraslow hemoglobin homotopic connectivity in the somatosensory cortex at 7 dpi. Average (mock, N=22, ZIKV, N=23) pixel-wise bilateral FC maps across mice for (a) infraslow hemoglobin and (c) delta calcium. Pixel-wise difference images (e) and two-sample t-test (left) and thresholded image (right) for P<0.01 by a cluster-based thresholding method for (f) delta calcium and (g) infraslow hemoglobin. Spatially averaged Pearson correlation value over the whole FOV for (b) delta calcium and (d) infraslow hemoglobin. Red horizontal bars represent the median value, and the box edges represent the interquartile range. Extending lines represent the maximum and minimum values. Significance determined by a two-sample t-test. Error bars are standard deviations and significance is determined by a two-sample t-test. $\mathrm{FOV}\approx 1.1\;{\mathrm{cm}}^{2}$.

Based on prior work using functional cortical connectivity (fc)-WFOI to assess cortical regions involved in learning and memory,^29^ we wondered if widefield optical imaging could detect a disruption in cortical networks involved in spatial navigation and memory, including those with connections to the retrosplenial cortex, an integrative hub for spatial cognition.^50^ To determine cerebral cortical connectivity during and after recovery from ZIKV encephalitis, we performed widefield optical imaging of Thy1-GCAMP6f mice that were i.c. mock- or ZIKV-Dak-MA-infected. Cranial window surgery and functional cortical imaging were performed at 7 dpi, which corresponds to peak encephalitis [Fig. 1(c)] and peak infection [Figs. 1(d)–1(g)]. Calcium dynamics produced by genetically encoded calcium indicators (GECIs) provide improved temporal resolution and more direct neural recording than downstream hemodynamics, whereas the latter provides an approximation for what the blood oxygen level-dependent (BOLD) fMRI signal might produce in human subjects.^22^ One measure of FC in the brain is homotopic connectivity, which measures FC of spatially mirrored locations in each hemisphere of the brain. Bilateral FC analysis [Fig. 2(a), 2(c) left] revealed high levels of homotopic connectivity in mock-infected mice using both infraslow^46^ (0.009 to 0.08 Hz) hemoglobin and delta (0.4 to 4.0 Hz) calcium frequency bands. The average bilateral FC value over the entire field of view (FOV) for both infraslow hemodynamics and delta calcium dynamics was significantly lower in ZIKV-infected mice [Figs. 2(b), 2(d)]. Cluster size-based statistical thresholding revealed an FC deficit using delta calcium dynamics that was constrained to somatosensory regions [Fig. 2(g)], whereas there was a more global decrease in FC strength using infraslow hemodynamics that included the retrosplenial cortex [Fig. 2(f)]. Overlay of the deficit in Fig. 2(g) onto an adapted Paxinos atlas-based cortical parcellation^25^ (Fig. S2 in the Supplementary Material) confirmed ZIKV-induced deficits in delta calcium bilateral FC clustered within somatosensory areas in addition to parietal and a small portion of the visual cortices. To confirm that the cortical needle insertion necessary for cranial infections would not artifactually result in FC differences, we compared bilateral FC in mice from the current study that were i.c. injected with mock solution 7 days prior to imaging and compared it with bilateral FC in mice that had received a peripheral injection 72 h prior to imaging (N=8) (Fig. S3 in the Supplementary Material). We found no differences in FC between mice who received i.c. compared with peripheral injection, which demonstrates that the intracranial needle insertion had no effect on FC measures.

### 3.4 Cortical Connectivity Improves After Recovery from ZIKV-Dak-MA

**Fig. 3:**
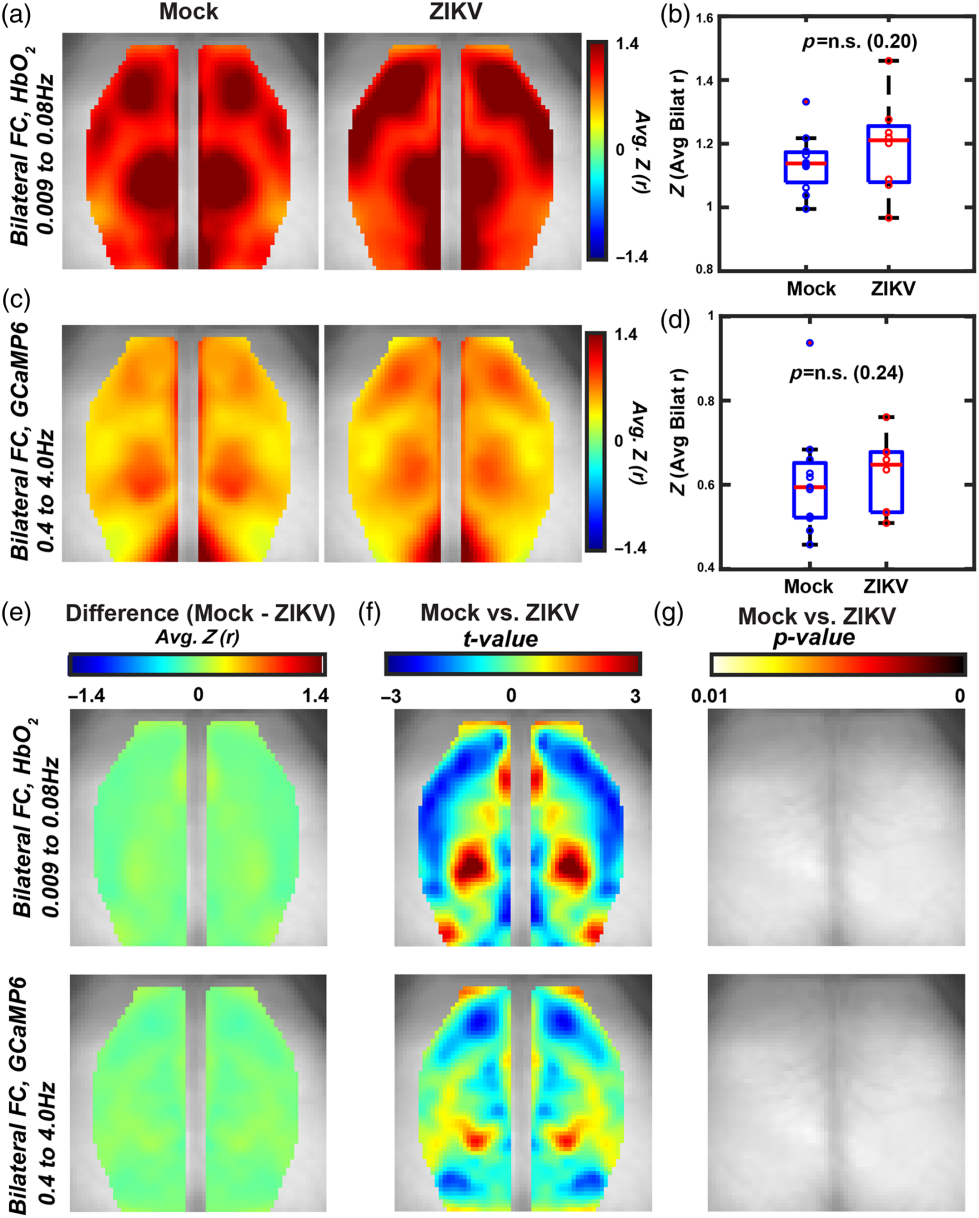
ZIKV-induced FC deficits resolve by 42 dpi. Average (mock, N=11, ZIKV, N=8) bilateral FC maps at 42 dpi for (a) infraslow hemoglobin and (c) delta calcium. Pixel-wise difference images (e) and two-sample t-test (left) and thresholded image (right) for P<0.01 by a cluster-based thresholding method for (f) delta calcium and (g) infraslow hemoglobin. Spatially averaged Pearson correlation value over the whole FOV for (b) delta calcium and (d) infraslow hemoglobin. Red horizontal bars represent the median value, and the box edges represent the interquartile range. Extending lines represent the maximum and minimum values. Significance determined by a two-sample t-test. Error bars are standard deviations and significance is determined by a two-sample t-test. The one data point extending beyond the maximum value is a statistical outlier in the mock group. No significance via a paired t-test (outlier removed). $\mathrm{FOV}\approx 1.1\;{\mathrm{cm}}^{2}$.

In humans and mice, cognitive impairments may persist long after recovery from acute ZIKV infection.^10^^,^^14^ Because ZIKV-Dak-MA-recovered mice have impaired spatial learning [Fig. 1(j)], we determined whether cortical connectivity networks measured by widefield optical imaging in recovered animals could detect alterations in cortical networks implicated in spatial learning via imaging of a cohort of mock- versus ZIKV-Dak-MA-infected mice at both 7 dpi and 42 dpi. Average (mock, N=11, ZIKV, N=8) paired bilateral FC maps using infraslow hemoglobin and delta calcium dynamics at 42 dpi revealed no difference between FC maps of mock- and ZIKV-infected mice at the 42 dpi recovery timepoint (Fig. 3). To determine if this lack of connectivity differences at 42 dpi was indicative of recovery of FC networks, we performed serial imaging in a subset of animals, imaging them at 7 dpi and then again at 42 dpi. Somatosensory deficits were detected at acute timepoints within a limited (N=8 ZIKV) dataset for delta calcium (Fig. S5 in the Supplementary Material), and a global FC deficit is present in the infraslow hemoglobin (Fig. S6 in the Supplementary Material) data at 7 dpi via cluster extent-based thresholding (Fig. S7 in the Supplementary Material), which were both similar to patterns seen in Fig. 2. To investigate ipsilateral connectivity patterns, we calculated FC using a seed-based approach with the delta calcium (Fig. S7 in the Supplementary Material) and infraslow hemoglobin (Fig. S8 in the Supplementary Material) data. The calcium data illustrated deficits in ipsilateral connectivity between somatosensory, visual, retrosplenial, and motor cortices. The hemoglobin data presented ipsilateral deficits in somatosensory, auditory, retrosplenial, parietal, and frontal cortices. By contrast, at 42 dpi no deficit in either infraslow hemoglobin and delta calcium dynamics remained at [Figs. 3(a), 3(b)]. The bilateral maps for each mouse at 42 dpi were spatially averaged across the whole FOV, as in Fig. 2, and displayed as boxplots for mock and ZIKV-recovered groups [Figs. 3(b), 3(d)], showing no significant differences between groups. In addition, there were no sex-dependent significant differences in average bilateral FC (Fig. S9 in the Supplementary Material) or delta power (Fig. S10 in the Supplementary Material) at 42 dpi.

### 3.5 Alterations in Cellular Content are Consistent with FC Changes

**Fig. 4:**
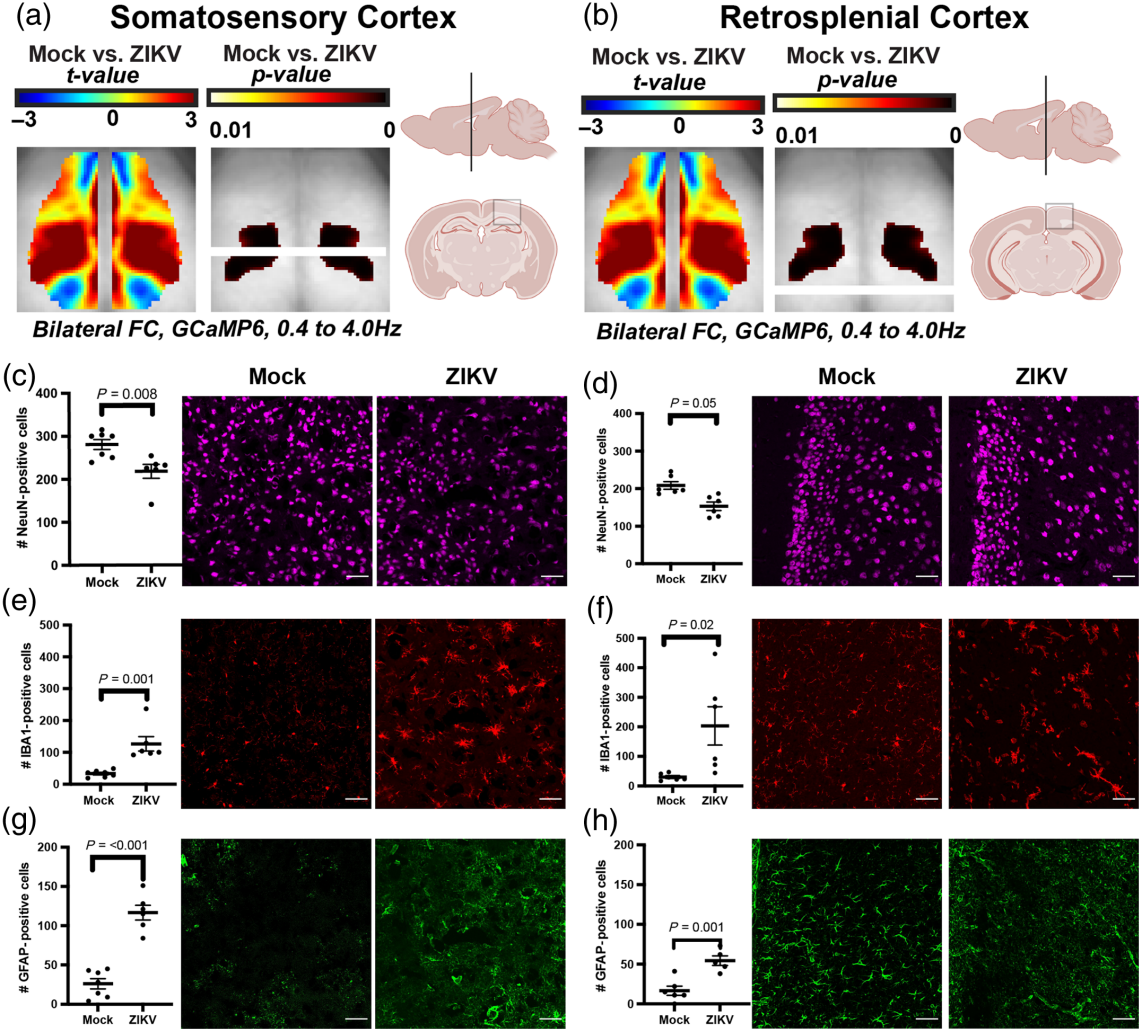
ZIKV infection induces cellular changes not only in the somatosensory cortex but also in a control region at 7 dpi. (a) Diagram showing the approximate location of somatosensory cortex samples in axial, sagittal, and coronal planes. (b) Diagram showing the approximate location of retrosplenial cortex samples in axial, sagittal, and coronal planes as reference regions. (c), (e), (g) NeuN (top), Iba1 (middle), and GFAP (bottom) staining of the somatosensory cortex, in which NeuN+ cells are significantly decreased, Iba1+ cells are significantly increased, and GFAP+ cells are increased in Zika-infected mice compared with mock mice. Mock, n=7; Zika-infected, n=6. Data were pooled from two independent experiments. (d), (f), (h) NeuN (top), Iba1 (middle), and GFAP (bottom) staining of the retrosplenial cortex, in which NeuN+ cells are significantly decreased, Iba1+ cells are significantly increased, and GFAP+ cells trend toward an increase in Zika-infected mice compared with mock mice, despite no associated difference in interhemispheric connectivity. Images taken at 20×. Mock, n=6; Zika-infected, n=6. Data were pooled from two independent experiments. Scale bars=50  μm. Analysis for panels (c)–(h) performed by paired t-test. Landmark figures in panels (a) and (b) created in BioRender.^51^

To better understand the cellular correlates of altered FC in the somatosensory cortex at 7 dpi, we examined neurons, astrocytes, and activated myeloid cells via IHC in the somatosensory cortex [Fig. 4(a)] and compared these with a control region, the retrosplenial cortex [Fig. 4(b)], which did not show significant changes in interhemispheric connectivity. Neuron numbers were decreased [Figs. 4(c), 4(d)] and GFAP+ [Figs. 4(e), 4(f)] and IBA1+ [Figs. 4(g), 4(h)] cells were increased in ZIKV-infected mice compared with mock-infected controls in both the somatosensory cortex and the retrosplenial cortex, although the statistical significance was larger in the somatosensory cortex. These findings indicate that cellular composition is associated with the FC measured by fast calcium dynamics [Figs. 4(a), 4(b)]. To further assess the relationship between FC and cellular composition in the somatosensory cortex at 7 dpi, a scatter plot was generated for Z(r) and quantified NeuN-, Iba1-, and GFAP-positive cells at 7 dpi [Figs. S11(A)–S11(C) in the Supplementary Material]. A Pearson correlation coefficient was then calculated for each relationship. There was a significant positive relationship between the number of Iba1-positive cells in the somatosensory cortex and the interhemispheric connectivity in the Zika-infected mice, r([3])=[0.906], p=0.012. The relationship between Z(r) and the number of Iba1-positive cells in the mock-infected mice was not significant, r([3])=[−0.313], p=0.609. The relationship between Z(r) and the number of GFAP-positive cells in both the mock- and ZIKV-infected mice was not significant (r([3])=[0.688], p=0.199 and r([3])=[−0.151], p=0.809, respectively). The relationship between Z(r) and the number of NeuN-positive cells in both the mock- and ZIKV-infected mice was not significant (r([3])=[−0.049], p=0.937 and r([3])=[0.058], p=0.926, respectively). To evaluate these associations between Iba1-positive cells and NeuN-positive cells qualitatively, data in Figs. 4(c), 4(e), 4(g) were reviewed for possible co-staining between Iba1 and NeuN and GFAP and NeuN. Multiple instances of Iba1 and NeuN and GFAP and NeuN costaining were identified in the cerebral cortex (Fig. S12 in the Supplementary Material). Similar findings were also observed in the hippocampus (not shown), suggesting that both microglia and astrocytes participate in phagocytosis of neurons during ZIKV infection.

### 3.6 Persistent Inflammatory Cells are Present in the Somatosensory Cortex During Recovery from ZIKV Encephalitis

**Fig. 5:**
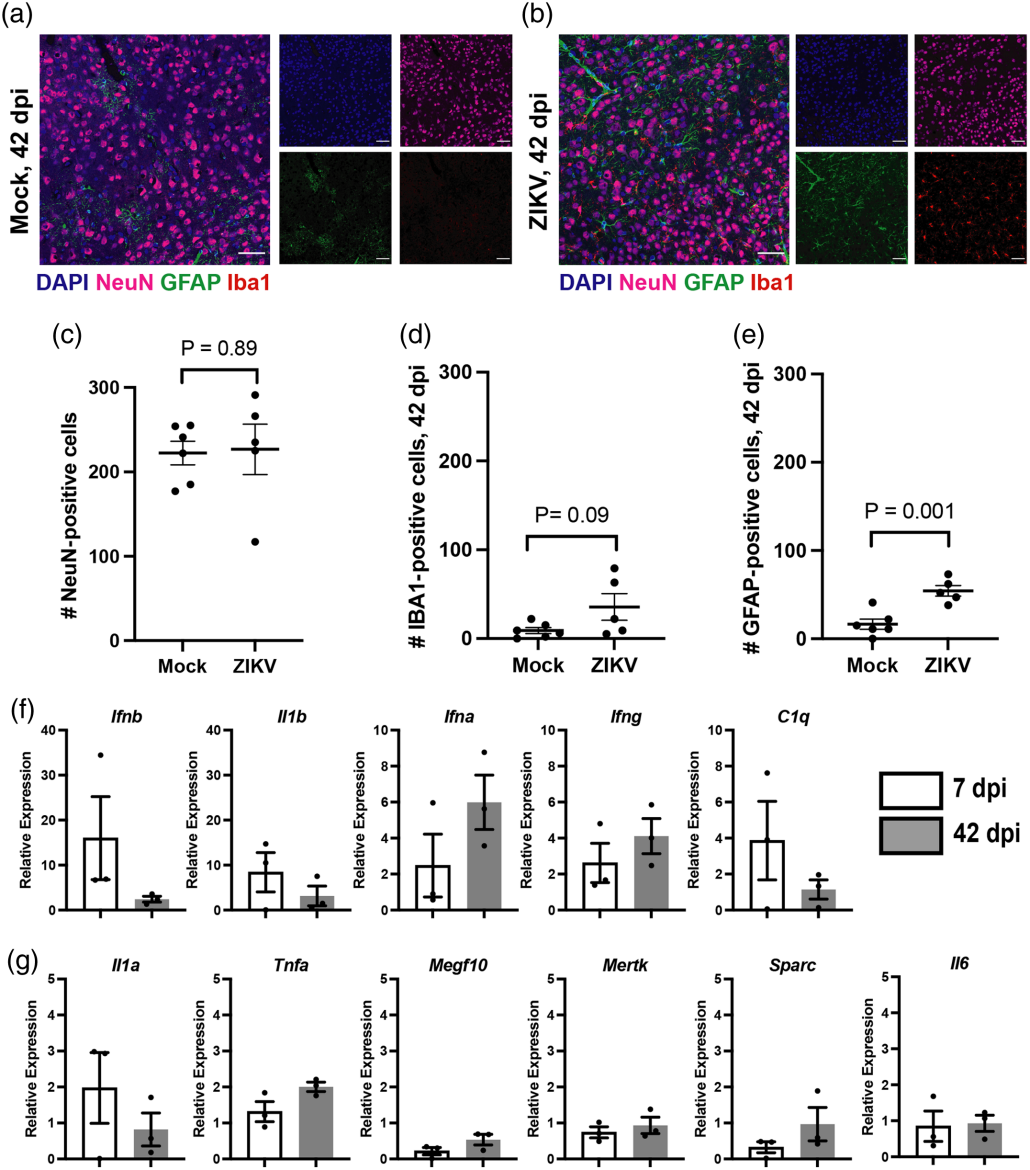
ZIKV infection resolves, but inflammation persists at 42 dpi. Staining of mock (a) and ZIKV-infected (b) tissue at 42 dpi from the somatosensory cortex. Quantification of NeuN (c), Iba1 (d), and GFAP (e) are also displayed. NeuN-positive cells appear to recover in ZIKV-infected mice, but Iba1-positive and GFAP-positive cells are increased compared with mock animals. N=6; Zika-infected, n=6. Data were pooled from two independent experiments. Paired t-test for statistical analysis in panels (c)–(e). Images taken at 20×. Scale bars=50  μm. Cytokine analysis of cerebral cortex comparing 7 dpi to 42 dpi (f), (g). Relative expression was calculated as $2^{-\Delta \Delta \mathrm{CT}}$ relative to GAPDH at 7 dpi or 42 dpi, respectively. Paired t-test was evaluated for each comparison in panels (f) and (g), and none reached statistical significance.

To determine whether recovery of interhemispheric connectivity (Fig. 3) was accompanied by a reduction in inflammation, brain tissues were harvested from mice immediately following widefield optical imaging on 42 dpi, followed by IHC detection of NeuN, Iba1, and GFAP within the somatosensory cortex. Although neuronal numbers in the somatosensory cortex of MA-recovered versus mock-infected mice were similar at 42 dpi [Fig. 5(a)], a trend toward higher levels of Iba1 and significantly higher GFAP persist [Figs. 5(b), 5(c)], indicating that cortical connectivity recovers, despite persistent inflammatory cells in the cortex. To determine if the persistently increased levels of cellular markers, particularly of Iba1+ and GFAP+ cells at 42 dpi (Fig. 5), were correlated with the recovery of FC, change in FC between 42 dpi and 7 dpi was calculated (ΔZ(r)) and compared with the cellular content at 42 dpi (Fig. S9 in the Supplementary Material). These analyses revealed that the numbers of Iba1+ and GFAP+ cells had strong linear relationships with changes in FC but did not reach significance (P=0.1169 and P=0.0577, respectively), indicating that the persistent presence of Iba1+ and GFAP+ cells at 42 dpi alone was not the etiology of FC recovery. An evaluation of cytokine expression was then performed using quantitative PCR and demonstrated that multiple pro-inflammatory cytokines in the cerebral cortex, including those known to mediate microglial and astrocyte-mediated phagocytosis of synapses and synaptic recovery in other animal models of CNS infection, such as Il1b and C1q,^44^^,^^52^ were elevated at 7 dpi and decrease to levels similar to those seen in mock-infected animals at 42 dpi [Figs. 5(f), 5(g)], suggesting a role for circulating cytokines in the health of functional brain networks.

### 3.7 Presynaptic Termini Loss During Acute Infection and Restoration During Recovery Underlie Alterations in FC

**Fig. 6:**
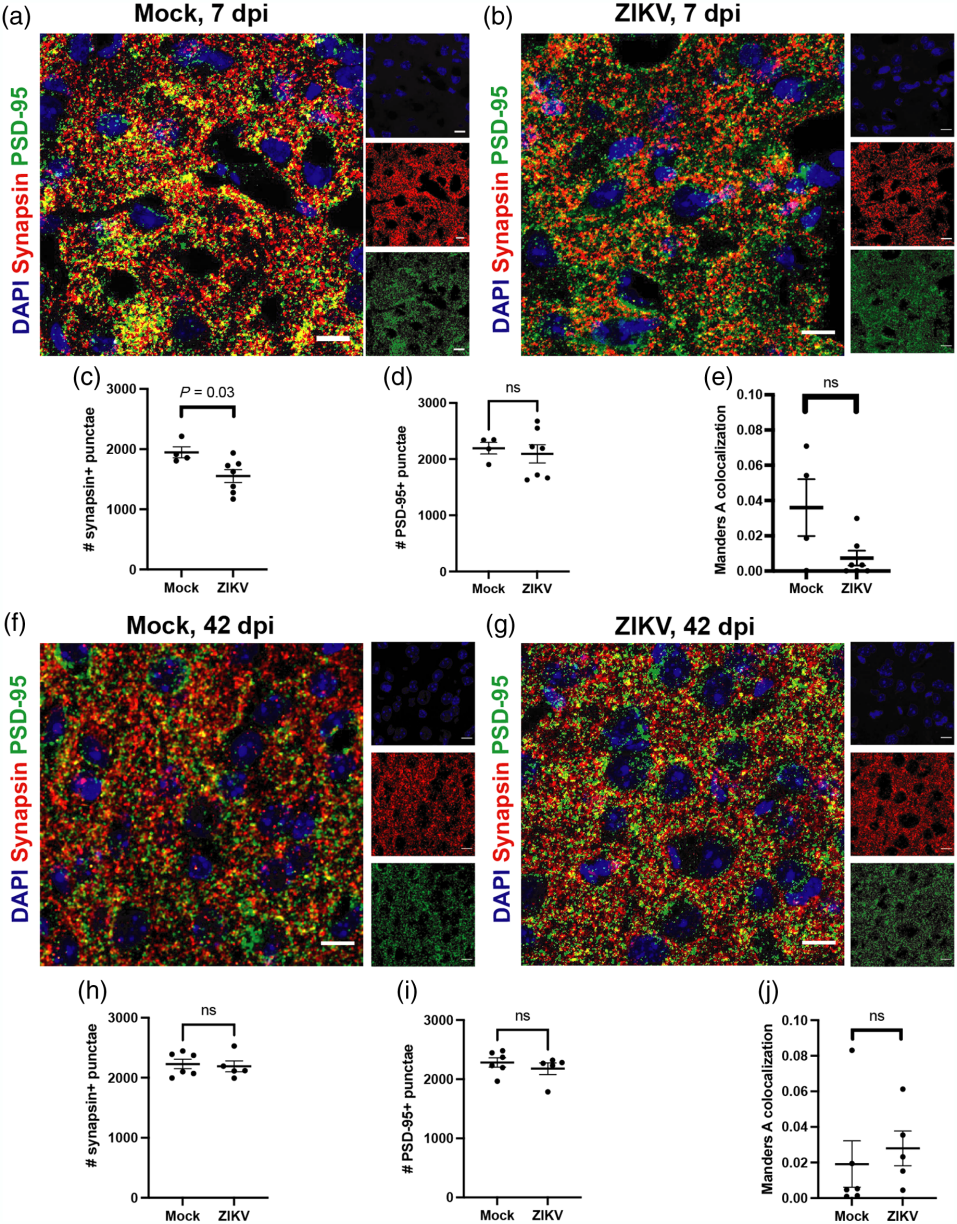
FC in ZIKV infection is associated with a decreased pre-synaptic marker, synapsin, during acute infection and recovered synapsin at 42 days post-infection. Representative synapse staining in the somatosensory cortex of (a) mock and (b) ZIKV-infected mice at 7 dpi. (c) Synapsin, a pre-synaptic marker, is decreased in the somatosensory cortex at 7 dpi. (d) PSD-95, a post-synaptic marker, is stable in mock and infected mice. (e) Colocalization of synapsin and PSD-95 is not significantly different. Images taken at 63×; scale bars: 10  μm. (d) Quantification of synapse markers showing mock (n=4) and Zika-infected (n=7) mice. Data was pooled from two independent experiments. Representative synapse staining in the somatosensory cortex of (f) mock and (g) ZIKV-infected mice at 7dpi. (h) Synapsin, a pre-synaptic marker, and PSD-95 (i), a post-synaptic marker do not differ between mock and infected mice after recovery. Images taken at 63×; scale bars: 50  μm. (j) Colocalization between pre- and post-synaptic markers is not different. Quantification of synapse markers showing mock (n=6) and Zika-infected (n=5) mice. Data was pooled from two independent experiments. Statistical analysis was performed by paired t-test.

Prior work has suggested that human resting-state FC networks are influenced by synapses.^53^ Therefore, we evaluated the expression of synaptic markers that are known to be ubiquitously expressed in the cerebral cortex at 7 dpi.^54^ Quantitative IHC detection of synapsin, a presynaptic marker, and PSD-95, a post-synaptic marker, within the somatosensory cortex, revealed significantly decreased detection of synapsin, but not PSD-95, in ZIKV-Dak-MA-infected mice compared with mock-infected controls [Figs. 6(a)–6(d)]. Colocalization of synapsin and PSD95 also showed a trend toward decrease in mice during acute infection compared with mock-infected controls [Fig. 6(e)], suggesting an association between synapsin+PSD-95+ synapses and interhemispheric connectivity during acute ZIKV infection.

Because presynaptic termini loss was associated with decreases in interhemispheric connectivity at 7 dpi, we similarly evaluated the expression of synapsin and PSD-95 in somatosensory cortex tissues derived from ZIKV-Dak-MA-recovered versus mock-infected animals at 42 dpi. In contrast to the decrease in presynaptic synapsin+ termini observed at 7 dpi, no differences in numbers of synapsin+ presynaptic termini or colocalization of synapsin and PSD-95 were observed between mock- and ZIKV-Dak-MA-infected animals [Figs. 6(f)–6(j)]. Despite the recovery of synapses and cortical connectivity in mice during recovery, hippocampal injury without neuronal recovery and spatial learning deficits persist at 42 dpi [Fig. 1(j)]. These data indicate that cortical networks, via restoration of synapses, can be restored even in the setting of severe subcortical injury. We also evaluated the relationship between FC and synapses as determined by Manders coefficient, a quantitation of overlap between synapsin and PSD95 punctae at both 7 dpi and 42 dpi. We found that while loss of the presynaptic marker, synapsin, was found to decrease and 7 dpi and recover at 42 dpi in the somatosensory cortex (Fig. 6). To determine if FC at 7 dpi was predictive of FC at 42 dpi, FC values within mouse were plotted against each other, and there were no significant correlations between the two time points, indicating that 7 dpi FC was not predictive of 42 dpi FC [Fig. S11(G) in the Supplementary Material]. Manders coefficient did not have a linear relationship with FC [Figs. S9(H) and S9(I) in the Supplementary Material]. When we compared FC in both mock- and ZIKV-MA-infected mice at 7 dpi and 42 dpi, we also noted that mock-infected mice had an overall trend toward decreased FC from 7 to 42 dpi, indicating a possible age-related change in FC (Fig. S13 in the Supplementary Material).

## 4 Discussion

Although the importance of immune cell infiltration and activation during acute viral infection is well-established, its effect on CNS recovery after viral clearance is poorly understood. In this study, we demonstrate that loss of cortical FC is associated with neuronal injury and synapse loss during acute ZIKV encephalitis. Via repeated FC assessment, we also determined that recovery of cortical FC is associated with a decrease in cytokine activation during the acute infection, suggesting that cytokine activation may play a role in the integrity of functional brain networks during and after viral infection. The use of MA-ZIKV, which causes severe injury of the hippocampal formation, also revealed decoupling between cortical FC recovery and hippocampal-based learning.

Recent FC research has uncovered the complexity of the acquired brain network data by demonstrating that functional networks reflect not only neuron health but also the health of synapses, glia, and microglia that support brain networks.^53^ In this study, we evaluated resting state cortical FC in conjunction with immunohistochemical analyses to determine if functional neuroimaging might correlate with neurocognitive findings as a noninvasive assessment of cognitive status during acute infection with, and recovery from, ZIKV encephalitis. In prior studies, the ZIKV-Dak strain has been shown to primarily target the hippocampus.^14^ Here, we selected a highly virulent, mouse-adapted strain of Zika-Dak (ZIKV-Dak-MA) for our study, which exhibits widespread infection throughout the brain, including the cerebral cortex. In the current study, the ZIKV-Dak-MA strain was also observed to induce severe destruction of the hippocampal formation. Consistent with this, we found that compared with mice infected with ZIKV-Dak, mice infected with ZIKV-Dak-MA were completely unable to learn a spatial memory task assessed via the Barnes maze. We wondered if widefield optical imaging could detect this persistent cognitive deficit as alterations in cortical FC. Although we found that cortical FC decreased in the somatosensory cortex during acute infection (7 dpi), it appeared to largely recover at 42 dpi, despite hippocampal obliteration.

We also performed immunohistochemical studies to correlate FC findings with significant alterations in inflammatory markers during acute ZIKV encephalitis. Our analyses revealed not only decreased neurons and increased GFAP- and Iba1-positive cells in the somatosensory cortex, but similar trends in the retrosplenial cortex, which did not exhibit contralateral FC deficits as calculated in Fig. 2. Furthermore, by expanding the FC analysis performed in Fig. 2 to include a seed-based approach in Figs. S7–S8 in the Supplementary Material, we note multiple ipsilateral and contralateral FC changes among the functional cortical regions shown. These data indicate these acute responses to infection correlated with FC findings. Similarly, we found that neuronal numbers did not differ between mock- and ZIKV-infected mice at 42 dpi, but the numbers of GFAP+ cells remained increased in the somatosensory cortex at 42 dpi. Similar to the inflammation signaled by increased GFAP+ cells at 42 dpi in the cortex, we detected persistent myeloid cell activation with increased Iba1-positive cells throughout the hippocampus at 42 dpi, and severe injury, with ablation of neurons in the CA3 region. There was also some suggestion by histochemical staining that both microglia and astrocytes may participate in neuronal phagocytosis, both at 7 dpi and 42 dpi. These data support our findings that i.c. ZIKV-Dak-MA-infected mice are unable to learn the Barnes maze after viral clearance and recovery. Nevertheless, these findings demonstrate that functional networks are driven by synaptic function during the course of ZIKV infection and recovery as measured by widefield optical imaging. This study also contributes to and supports the growing body of literature to suggest contributions of neurons, glia, microglia, and synapses to resting state-FC networks^53^^,^^55^^–^^57^ and that anti-viral, pro-inflammatory cytokines may facilitate some of these interactions.^44^^,^^52^

In addition to the primary findings of the study, we also found high average delta power in the Zika-infected mice at 7 dpi, which returned to levels close to that of mock-infected mice at 42 dpi. Previous studies have shown that increased Delta power is correlated with poor outcomes in patients with stroke, for example,^58^ and this is directly related to neuronal metabolism. This indicates that delta power is also inversely related to the health of brain networks and provides different information about cognition from that found in interhemispheric connectivity measures. This would be consistent with our findings that mice infected with ZIKV do, in fact, have long-term cognitive sequelae, even though their interhemispheric cortical connectivity recovers. Future work could explore this by measuring delta power simultaneously with a quantitative electroencephalogram.

In addition, we included both infraslow hemoglobin mapping and delta calcium mapping in our FC analysis. Although our instrumentation allows both signals to be collected simultaneously, they are still mapping distinct neurological processes in very different frequency bands and are subject to distinct confounding variables. This is best illustrated by the differences in connectivity maps seen in Fig. 2 between hemoglobin and calcium maps. When comparing results within Fig. 2(e), the locus of the biggest connectivity change is focused on the somatosensory and parietal regions for both hemoglobin and calcium. An additional possible explanation is that neural calcium and vascular hemoglobin respond differently to immune system activation by ZIKV. Another possibility is that ZIKV alters neurovascular coupling, which could be explored in future studies.

The findings of this study may have some limitations. We found that when we repeatedly imaged the same mice at 7 and 42 dpi, not only did ZIKV-infected mice exhibit similar FC compared with mock-infected mice at 42 dpi, but mock-infected mice demonstrated a decrease in FC from 7 to 42 dpi. As mice were infected and imaged as young adults (8–9 weeks old), and imaged again at 42 dpi (15 weeks old), it is possible that aging contributed to our findings at the recovery time point. Previous studies have shown that connectivity does change over the lifespan,^59^^,^^60^ but there is little functional neuroimaging data evaluating the ages used in our study. This may be an area for future investigation.

Although excellent two-dimensional spatial resolution and time-series data are captured with widefield optical imaging, another known limitation of widefield optical imaging is the depth of resolution due to the effects of light scattering and absorption. Our model, however, showed recovery of cortical FC occurs despite severe hippocampal injury shown by both immunohistochemical and behavioral approaches. Thus, our aim to determine if FC as measured by WFOI could predict neurocognitive deficits was limited by the recovery of cortical networks in the setting of subcortical brain injury. Although our results suggest that WFOI can detect the effects of viral infection and associated immune response and inflammation on cortical brain networks during acute viral encephalitis, future work is needed to determine the limits of detection of alterations in cortical-subcortical networks by WFOI.

In summary, our data demonstrate that cortical FC decreases during acute ZIKV encephalitis due to loss of presynaptic termini, which improves after recovery from infection. This connectivity and synapse recovery correlated with the degree of myeloid cell activation and was associated with increased in antiviral, pro-inflammatory cytokine expression at 7 dpi with a nearly return to baseline cytokine levels at 42 dpi, implicating neuroinflammation in the recovery of cognitive networks in the brain after ZIKV infection. We also found that persistent astrocyte activation was anticorrelated with FC. This data has important implications for immunomodulatory therapies in post-infectious neurocognitive recovery, as it suggests that not all neuroinflammation is detrimental. For example, our data suggest that IL-1α/β and C1q are elevated during acute infection, and they have both been implicated in virally-mediated cognitive deficits previously.^41^^,^^44^^,^^52^ Furthermore, IFN-α, IFN-γ, and TNF-α show a trend toward being increased during recovery from infection. Specific immunomodulatory therapies that target each of these cytokines are clinically available, but future studies are needed to better understand the ideal timing of treatment as well as the potential off-target effects of these treatments. In addition, there is a role for understanding the individual contributions of myeloid cells and astrocytes in cortical synaptic recovery as demonstrated by the persistent Iba1 and GFAP staining during recovery during our study. Sparc, which is expressed by astrocytes, also showed a trend toward increased expression during recovery. Therefore, future studies will also be needed to understand the potential risks and benefits of targeting the production or function of myeloid cells and astrocytes. In sum, this study contributes an assay workflow for understanding the impact of infection and inflammation on the health of functional brain networks as well as opening future directions for the evaluation of immunomodulatory therapies in the setting of inflammatory brain processes.

## Supplementary Material



## Data Availability

The data that support the findings of this paper are not publicly available. They can be requested from the corresponding author at rklein8@uwo.ca
