# Supplementary material for: Zika virus encephalitis causes transient reduction of functional cortical connectivity
Source: Neurophotonics. 2024 Nov 28;12(Suppl 1):S14603. doi: 10.1117/1.NPh.12.S1.S14603 (PMC11603678; doi:10.1117/1.NPh.12.S1.S14603)
Supplement: Supplementary file 1 [file NPh_012_S14603_SD001.pdf]

Table S1. Statistical analyses for all tissue-based experiments.

Table S1. Statistical analyses for all tissue-based experiments.

FIGURE	VARIABLE	COMPARISON	DATA STRUCTURE	STATISTICAL TEST	OUTPUT	95% Confidence Interval	Cohen's d (interpretation)
4C	Number of NeuN+ cells, SSC, 7 dpi	Mock vs. ZIKV	normal	Unpaired t-test	t(11) = 3.235, p = 0.008	[20.03, 105.3]	1.78 (large effect size)
4D	Number of NeuN+ cells, RSC, 7 dpi	Mock vs. ZIKV	normal	Unpaired t-test	t(11) = 3.675, p = 0.004	[-83.98, -21.07]	2.05 (large effect size)
4E	Number of Iba1+ cells, SSC, 7 dpi	Mock vs. ZIKV	normal	Unpaired t-test	t(11) = 4.376, p = 0.001	[11.91, 105.8]	2.33 (large effect size)
4F	Number of Iba1+ cells, RSC, 7 dpi	Mock vs. ZIKV	normal	Unpaired t-test	t(11) = 2.411, p = 0.03	[13.35, 293.6]	1.40 (large effect size)
4G	Number of GFAP+ cells, SSC, 7 dpi	Mock vs. ZIKV	normal	Unpaired t-test	t(11) = 8.128, p < 0.001	[66.05, 115.1]	4.47 (large effect size)
4H	Number of GFAP+ cells, RSC, 7 dpi	Mock vs. ZIKV	normal	Unpaired t-test	t(11) = 0.5759, p = 0.58	[-43.40, 74.16]	0.82 (moderate effect size)
5C	Number of NeuN+ cells, SSC, 42 dpi	Mock vs. ZIKV	normal	Unpaired t-test	t(9) = 0.1439, p = 0.89	[-65.72, 74.72]	0.08 (small effect size)
5D	Number of Iba1+ cells, SSC, 42 dpi	Mock vs. ZIKV	normal	Unpaired t-test	t(9) = 1.901, p = 0.09	[-5.055, 58.26]	1.09 (large effect size)
5E	Number of GFAP+ cells, SSC, 42 dpi	Mock vs. ZIKV	normal	Unpaired t-test	t(9) = 4.536, p = 0.001	[18.915, 56.55]	2.75 (large effect size)
5F	Ifnb RNA expression relative to gapdh	7 dpi vs. 42 dpi	normal	Unpaired t-test	t(4) = 1.470, p = 0.28	[-12.08, 39.25]	1.12 (large effect size)
	Il1b RNA expression relative to gapdh	7 dpi vs. 42 dpi	normal	Unpaired t-test	t(4) = 1.470, p = 0.28	[-1.855, 4.161]	0.87 (moderate effect size)
	Ifna RNA expression relative to gapdh	7 dpi vs. 42 dpi	normal	Unpaired t-test	t(4) = 1.522, p = 0.20	[-2.896, 9.922]	1.24 (large effect size)
	Ifng RNA expression relative to gapdh	7 dpi vs. 42 dpi	normal	Unpaired t-test	t(4) = 1.020, p = 0.37	[-2.572, 5.558]	0.83 (moderate effect size)
	C1q RNA expression relative to gapdh	7 dpi vs. 42 dpi	normal	Unpaired t-test	t(4) = 1.209, p = 0.34	[-3.522, 8.954]	0.98 (moderate effect size)
5G	Il1a RNA expression relative to gapdh	7 dpi vs. 42 dpi	normal	Unpaired t-test	t(4) = 1.064, p = 0.37	[-1.855, 4.161]	0.869 (moderate effect size)
	Tnfa RNA expression relative to gapdh	7 dpi vs. 42 dpi	normal	Unpaired t-test	t(4) = 2.238, p = 0.12	[-0.1662, 1.546]	1.827 (large effect size)
	Megf10 RNA expression relative to gapdh	7 dpi vs. 42 dpi	normal	Unpaired t-test	t(4) = 1.766, p = 0.16	[-0.1830, 0.8230]	1.44 (large effect size)
	Mertk RNA expression relative to gapdh	7 dpi vs. 42 dpi	normal	Unpaired t-test	t(4) = 0.52, p = 0.52	[-0.5678, 0.9544]	0.564 (small effect size)
	Sparc RNA expression relative to gapdh	7 dpi vs. 42 dpi	normal	Unpaired t-test	t(4) = 1.317, p = 0.30	[-0.2117, 1.498]	1.08 (large effect size)
	Il6 RNA expression relative to gapdh	7 dpi vs. 42 dpi	normal	Unpaired t-test	t(4) = 0.1676, p = 0.88	[-1.245, 1.406]	0.137 (small effect size)
6C	Number of synapsin+ punctae, 7 dpi	Mock vs. ZIKV	normal	Unpaired t-test	t(9) = 2.482, p = 0.03	[-752.5, -34.89]	1.658 (large effect size)
6D	Number of PSD-95+ punctae, 7 dpi	Mock vs. ZIKV	normal	Unpaired t-test	t(9) = 0.68, p = 0.68	[-626.6, 426.0]	0.296 (small effect size)
6E	Manders A colocalizaion: synapsin+ plus PSD95+, 7 dpi	Mock vs. ZIKV	normal	Unpaired t-test	t(9) = 2.204, p = 0.05	[-0.0580, 0.0007]	1.184 (large effect size)
6H	Number of synapsin+ punctae, 42 dpi	Mock vs. ZIKV	normal	Unpaired t-test	t(9) = 1.226, p = 0.25	[-368.1, 109.4]	0.7522 (large effect size)
6I	Number of PSD-95+ punctae, 42 dpi	Mock vs. ZIKV	normal	Unpaired t-test	t(9) = 0.8382, p = 0.42	[-385.4, 177.0]	0.5026 (moderate effect size)
6J	Manders A colocalizaion, synapsin+ plus PSD95+, 42 dpi	Mock vs. ZIKV	normal	Unpaired t-test	t(9) = 0.5181, p = 0.62	[-0.0295, 0.0471]	0.3199 (small effect size)
S1C	Number of NeuN+ cells, CA3, 7 dpi	Mock vs. ZIKV	normal	Unpaired t-test	t(10) = 1.413, p = 0.19	[-45.53, 10.19]	0.5950 (moderate effect size)
S1D	Number of Iba1+ cells, CA3, 7 dpi	Mock vs. ZIKV	normal	Unpaired t-test	t(10) = 1.870, p = 0.09	[-15.32, 175.3]	1.080 (large effect size)
S1E	Number of GFAP+ cells, CA3, 7 dpi	Mock vs. ZIKV	normal	Unpaired t-test	t(10) = 1.174, p = 0.27	[-30.82, 99.49]	0.3773 (small effect size)
S1H	Number of NeuN+ cells, CA3, 42 dpi	Mock vs. ZIKV	normal	Unpaired t-test	t(9) = 0.6357, p = 0.54	[-47.42, 84.49]	0.3659 (small effect size)
S1I	Number of Iba1+ cells, CA3, 42 dpi	Mock vs. ZIKV	normal	Unpaired t-test	t(9) = 0.571, p = 0.03	[12.91, 201.8]	1.469 (large effect size)
S1J	Number of GFAP+ cells, CA3, 42 dpi	Mock vs. ZIKV	normal	Unpaired t-test	t(9) = 2.463, p = 0.04	[7.652, 180.0]	1.413 (large effect size)
S13	Z(r)	7 dpi mock vs. 42 dpi mock	normal	Two way ANOVA with Tukey's correction for multiple comparisons	p = 0.1889	[-0.03089, 0.2195]	
		7 dpi mock vs. 7 dpi zikv			p = 0.1873	[-0.03472, 0.2487]	
		7 dpi mock vs. 42 dpi zikv			p = 0.4087	[-0.06058, 0.2229]	
		42 dpi mock vs. 7 dpi zikv:			p = 0.9946	[-0.1290, 0.1544]	
		42 dpi mock vs. 42 dpi zikv:			p = 0.9939	[-0.1549, 0.1286]	
		7 dpi zikv vs. 42 dpi zikv:			p = 0.9615	[-0.1727, 0.121]	

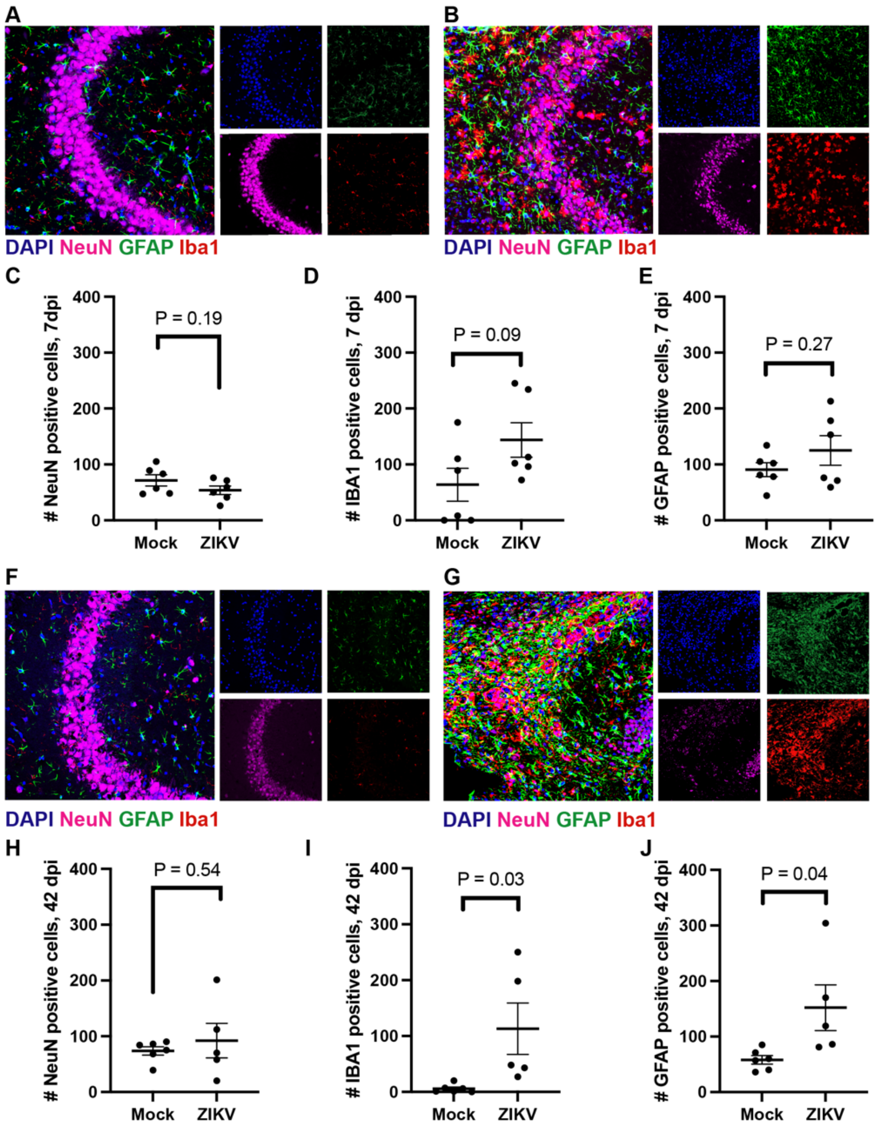

Figure S1. ZIKV infection is associated with cellular changes in the hippocampus during acute infection and severe hippocampal injury after recovery from infection. Representative CA3 regions from mock (A) and ZIKV-infected (B) mice at 7 dpi. There is a trend toward increased numbers of Iba1+ (D) and GFAP+ cells in the CA3 at 7 dpi. At 42 dpi, representative images of the mock-infected CA3 (F) compared to ZIKV-infected (G) CA3 shows severe injury to the ZIKV-infected CA3. Numbers of NeuN+ neurons are similar (H), and there are significant persistent elevations in the numbers of Iba1+ (I) and GFAP+ (J) cells at 42 dpi. Analysis performed by paired t-test.

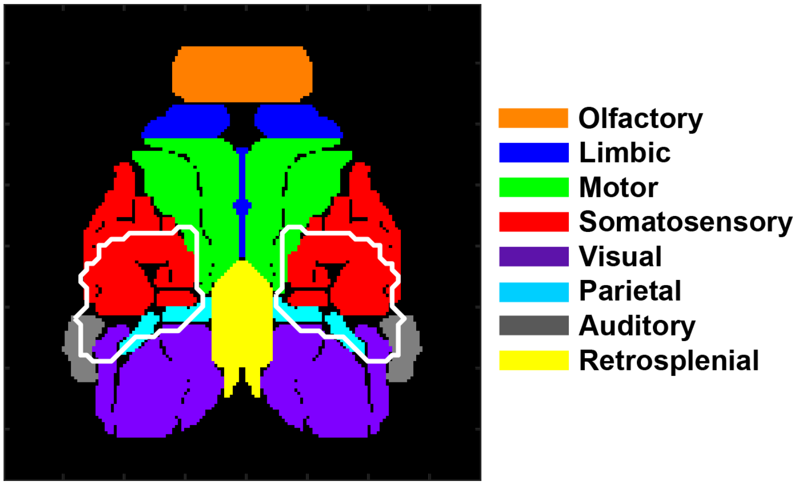

Figure S2. FC deficits align most strongly with the somatosensory cortex. Cortical Paxinos atlas parcellation adapted by White et al.[20] were used as a mapping template to identify anatomy associated with FC deficits. Using average FC maps of ZIKV-infected mice from Figure 1, FC deficit is outlined in white.

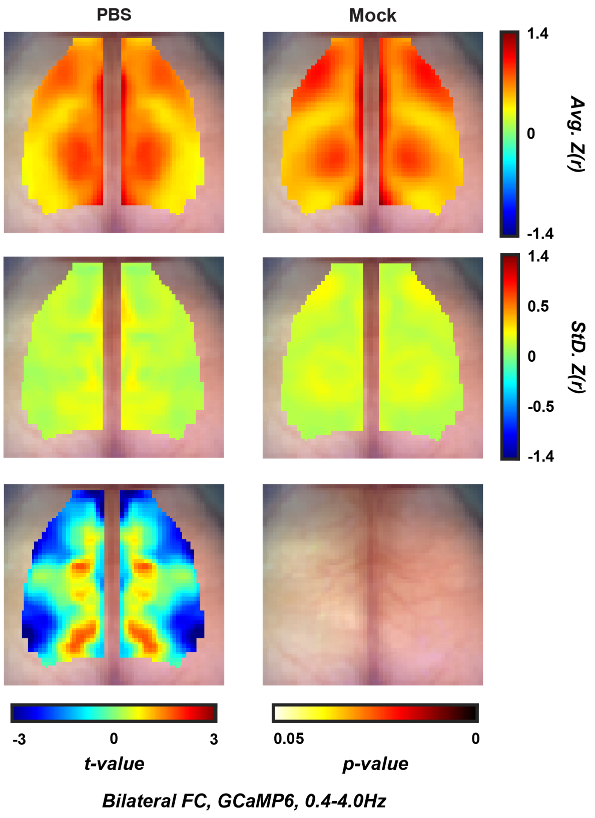

Figure S3: Mock infected FC is relatively unaffected by cranial needle injection. Average pixel-wise (PBS N=8, mock N=22) bilateral correlation maps (top row) and standard deviations (middle row) across mice. Pixel-wise two-sample *t*-test (bottom row, left) and threshold image (bottom row, right) for $p<0.01$ by a cluster-based thresholding method.

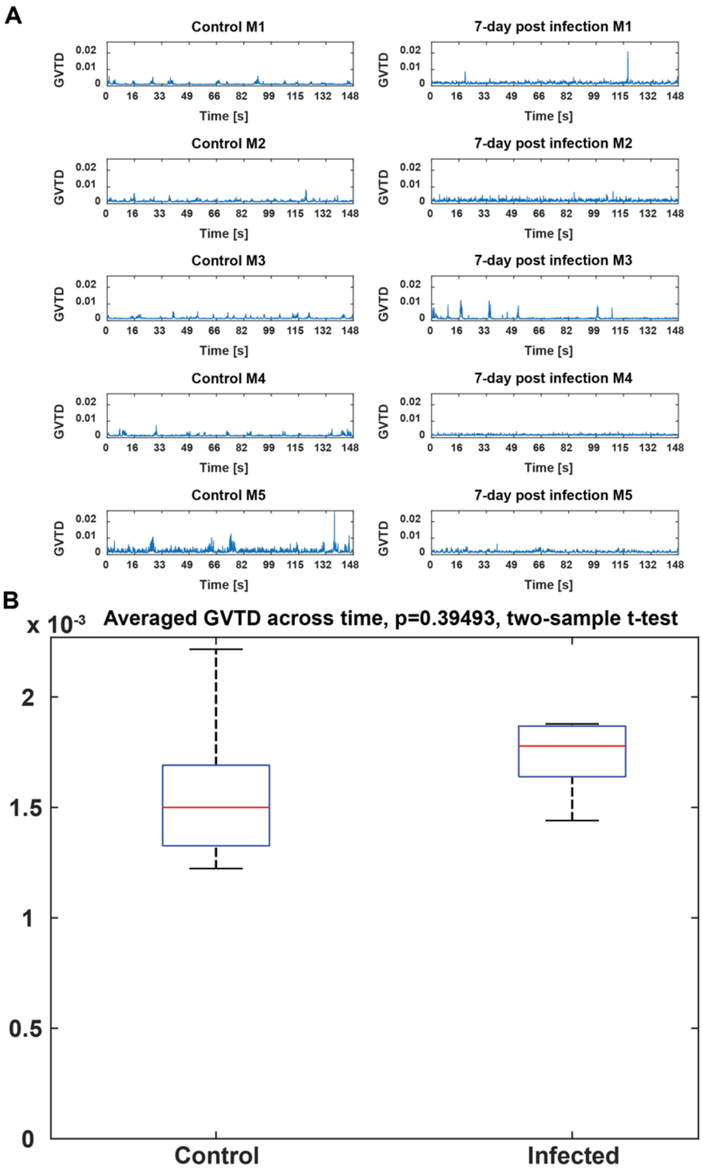

Figure S4: Global variation in temporal derivatives are not different between mock and ZIKV-infected mice at 7 dpi. (A) Raw GVTD plots for 5 mock and 5 ZIKV-infected mice at 7 dpi. (B) Averaged GVTD for all datasets in (A). There is not a statistically significant difference in GVTD between mock- and ZIKV-infected mice.

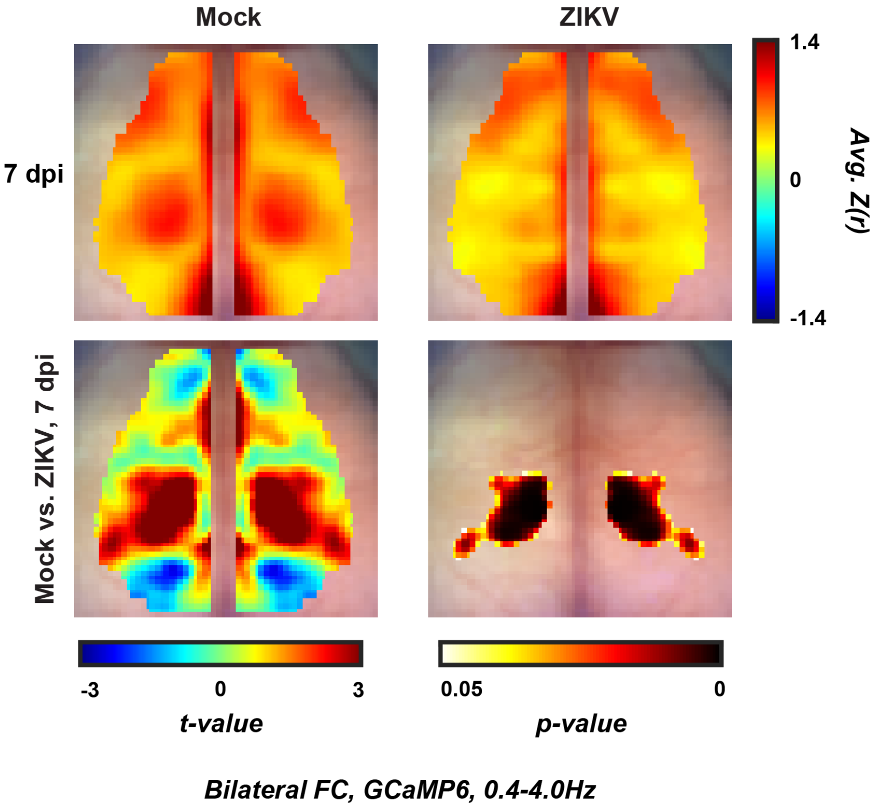

Figure S5: A smaller ZIKV sample size yields the same somatosensory based FC deficit at 7dpi in delta calcium. (Top row) Average (mock, N=11, ZIKV, N=8) pixel-wise bilateral FC maps across mice. (Bottom row) Pixel-wise two-sample *t*-test (left) and thresholded image (right) for $p<0.01$ by a cluster-based thresholding method.

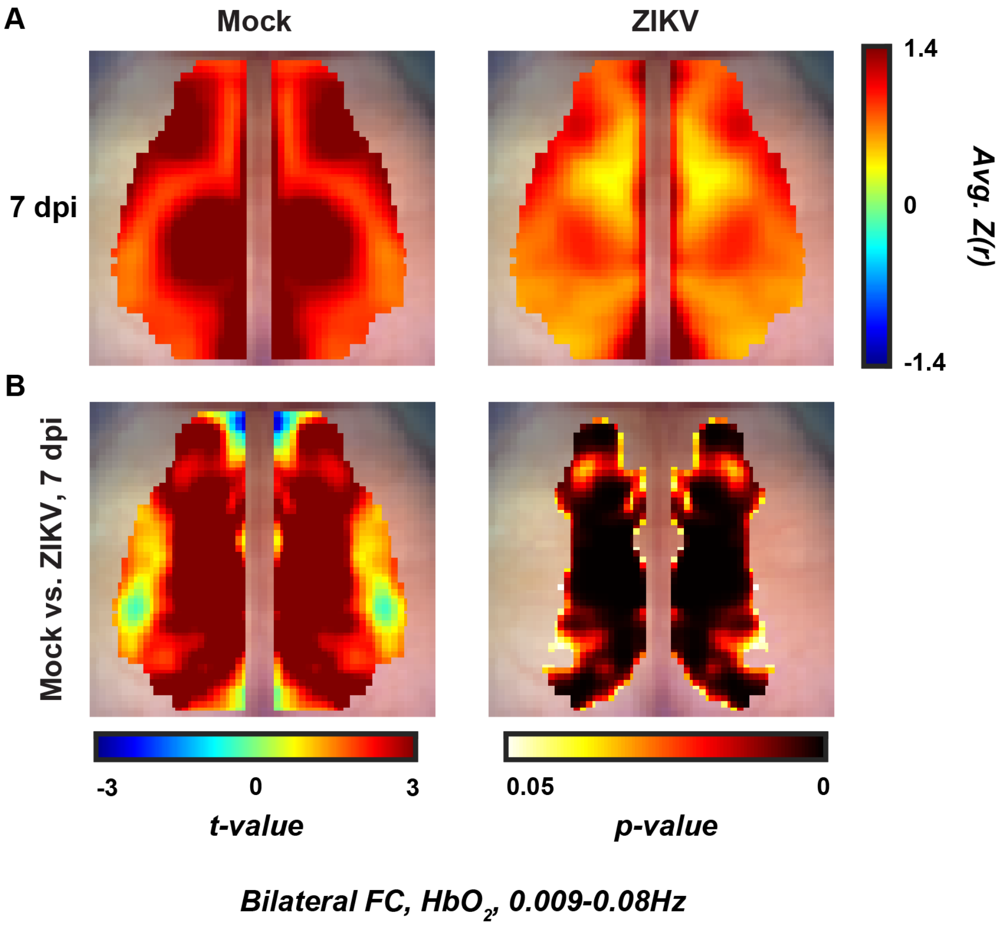

Figure S6: A smaller ZIKV sample size yields the same global FC deficit at 7dpi in infraslow hemoglobin. (Top row) Average (mock, N=11, ZIKV, N=8) pixel-wise bilateral FC maps across mice. (Bottom row) Pixel-wise two-sample *t*-test (left) and thresholded image (right) for $p<0.01$ by a cluster-based thresholding method.

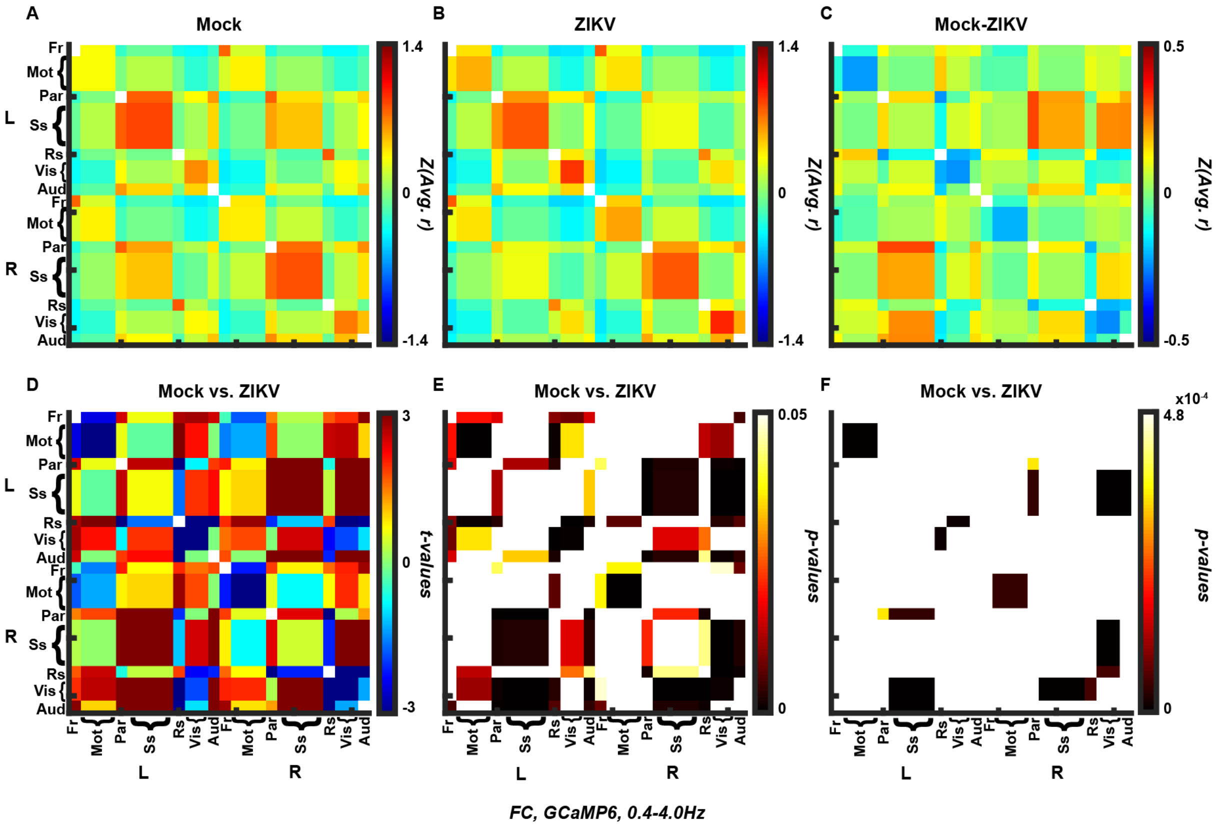

Figure S7. ZIKV infection weakens multiple network connections using calcium delta dynamics. (A,B) Average (mock N=22, ZIKV N=23) FC matrices displaying the Pearson correlation between network seeds shown on the x and y-axis, as well as the (C) difference in Pearson correlation between the mock and ZIKV matrices. (D) Network-wise two-sample *t*-test, and (E) corresponding result highlighting regions with a *p*-value<0.05 (uncorrected for multiple comparisons). (F) Matrices are thresholded to display *p*-values below the Bonferroni threshold for significance (two-sample *t*-test).

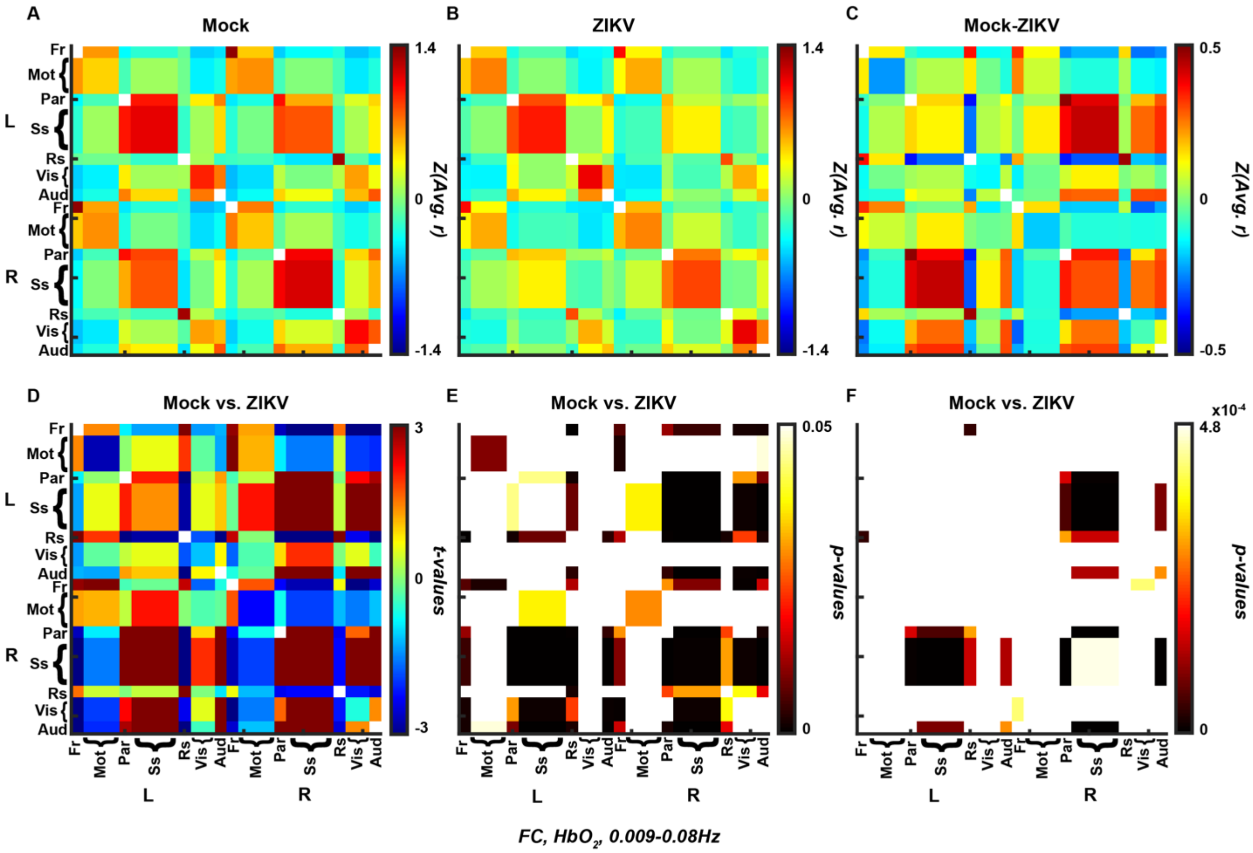

Figure S8. ZIKV infection weakens multiple network connections using hemoglobin infraslow dynamics. (A,B) Average (mock N=22, ZIKV N=23) FC matrices displaying the Pearson correlation between network seeds shown on the x and y-axis, as well as the (C) difference in Pearson correlation between the mock and ZIKV matrices. (D) Network-wise two-sample *t*-test, and (E) corresponding result highlighting regions with a *p*-value<0.05 (uncorrected for multiple comparisons). (F) Matrices are thresholded to display *p*-values below the Bonferroni threshold for significance (two-sample *t*-test).

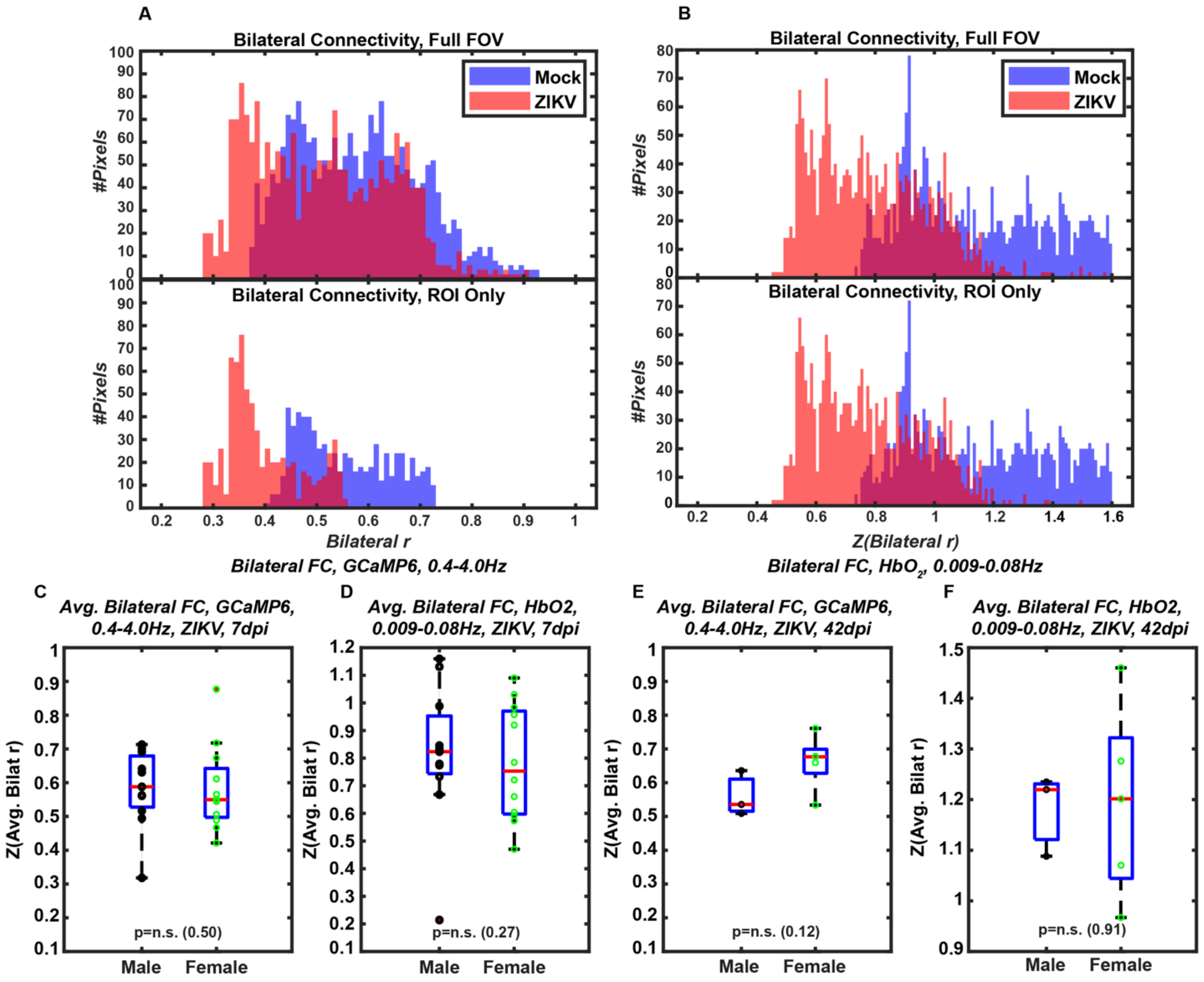

Figure S9. ZIKV infection effects hemodynamics more than calcium, in a sex-independent manner. Histogram of Pearson correlation values in the average bilateral FC maps from Figure 2 showing the whole FOV (top) and only the regions in the cluster-defined ROI (bottom) for (A) delta calcium and (B) infraslow hemoglobin. Average (mock N=22, ZIKV N=23) bilateral FC over the FOV separated by sex at 7dpi for (C) delta calcium and (D) infraslow hemoglobin. Average (mock N=11, ZIKV N=8) bilateral FC over the FOV separated by sex at 42dpi for (C) delta calcium and (D) infraslow hemoglobin. Significance testing by a two-sample t-test.

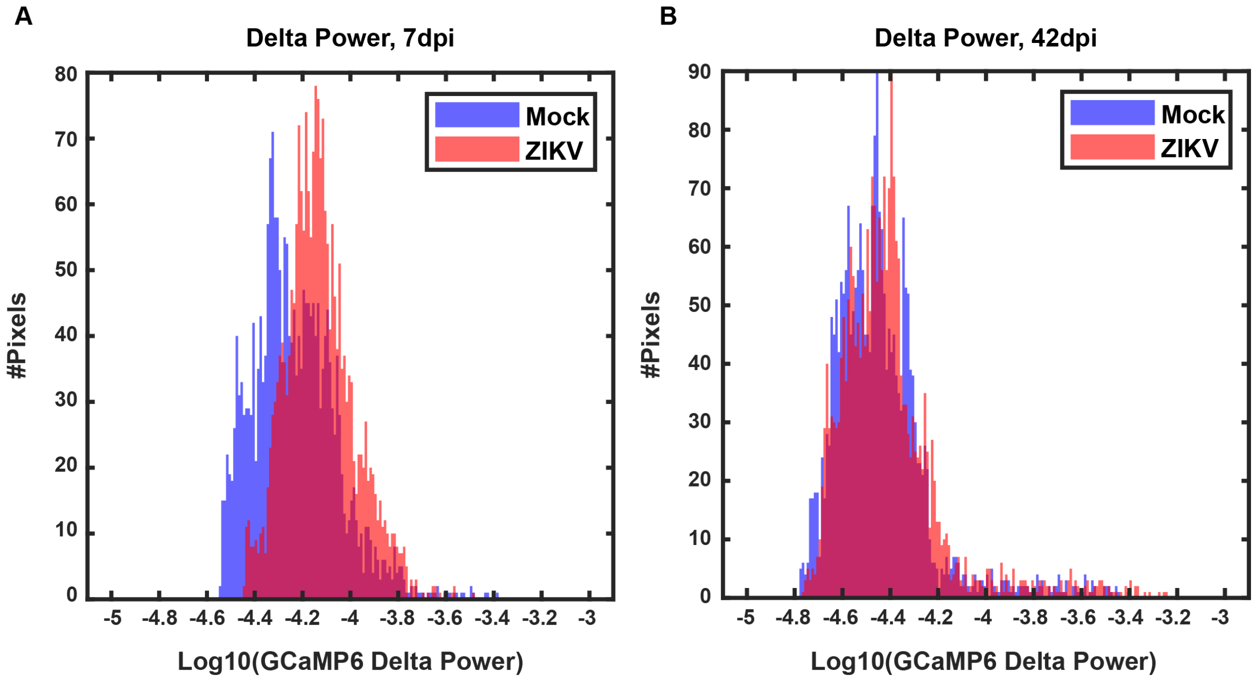

Figure S10. Acute ZIKV increases global delta power. Histogram displaying pixel-wise GCaMP6 delta power across the whole FOV at 7 dpi (left) and 42 dpi (right). Delta power increased during acute infection and recovers to healthy delta power after recovery from ZIKV infection. No statistical analysis performed; histograms for illustrative purposes only.

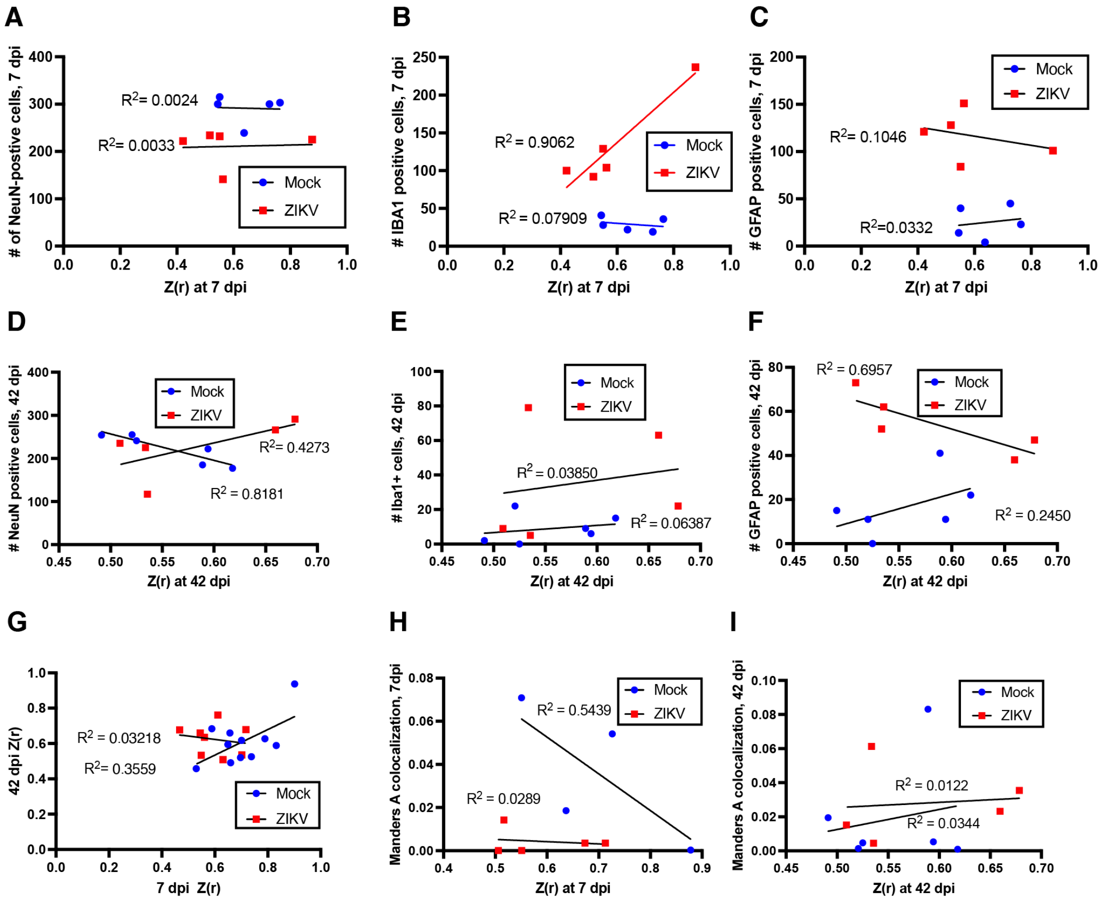

Figure S11. FC at 7 dpi does not predict FC at 42 dpi, but myeloid and astroglial activation does. Scatter plots of cellular content at 42 dpi vs. the within-mouse change in FC between acute infectiton and recovery, ΔZ(r), were calculated for NeuN+, Iba1+, and GFAP+ cells. A Pearson correlation coefficient was then calculated for each relationship. There were no significant positive relationships between the cellular content in the somatosensory cortex and the change in interhemispheric connectivity in mock or ZIKV-infected mice. However, there were suggestions of correlations between the improvement in ΔZ(r) and Iba1+ and GFAP+ cells at 42. The relationship between Iba1+ cells and ΔZ(r) in mock-infected mice was r([4])=[0.155], p = 0.7693 and in Zika-infected mice, r([3]) = [0.6138], p = 0.1169. The relationship between GFAP+ cells and ΔZ(r) in mock-infected mice was r([4])=[-0.2369], p = 0.6513 and in Zika-infected mice, r([3]) = [-0.8660], p = 0.0577. The relationship between NeuN+ cells and ΔZ(r) in mock-infected mice

was r([4])=[-0.1783], p = 0.7354 and in Zika-infected mice, r([3]) = [0.6656], p = 0.2201. Scatter plot of FC at 7 dpi vs. FC at 42 dpi (D) and Manders A colocalization, a measure of overlap of pre- and post-synaptic punctae, vs. FC were also calculated at 7 dpi (E) and 42 dpi (F) but did not reveal high correlation.

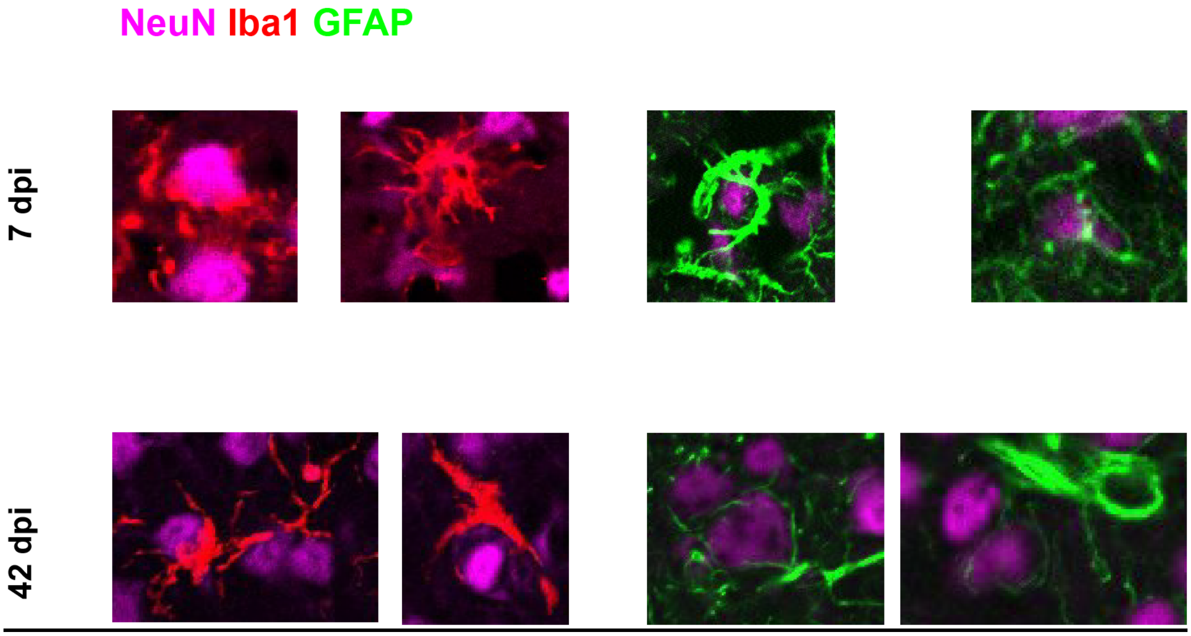

Figure S12. Colocalization of myeloid cells and astrocytes with neurons. Histochemical staining of NeuN (magenta), Iba1 (red) and GFAP (green) positive cells, demonstrating colocalization, or overlap, between NeuN and Iba1 and GFAP and NeuN at both 7 dpi and 42 dpi. This suggests possible phagocytosis of neurons by CNS myeloids cells and astrocytes.

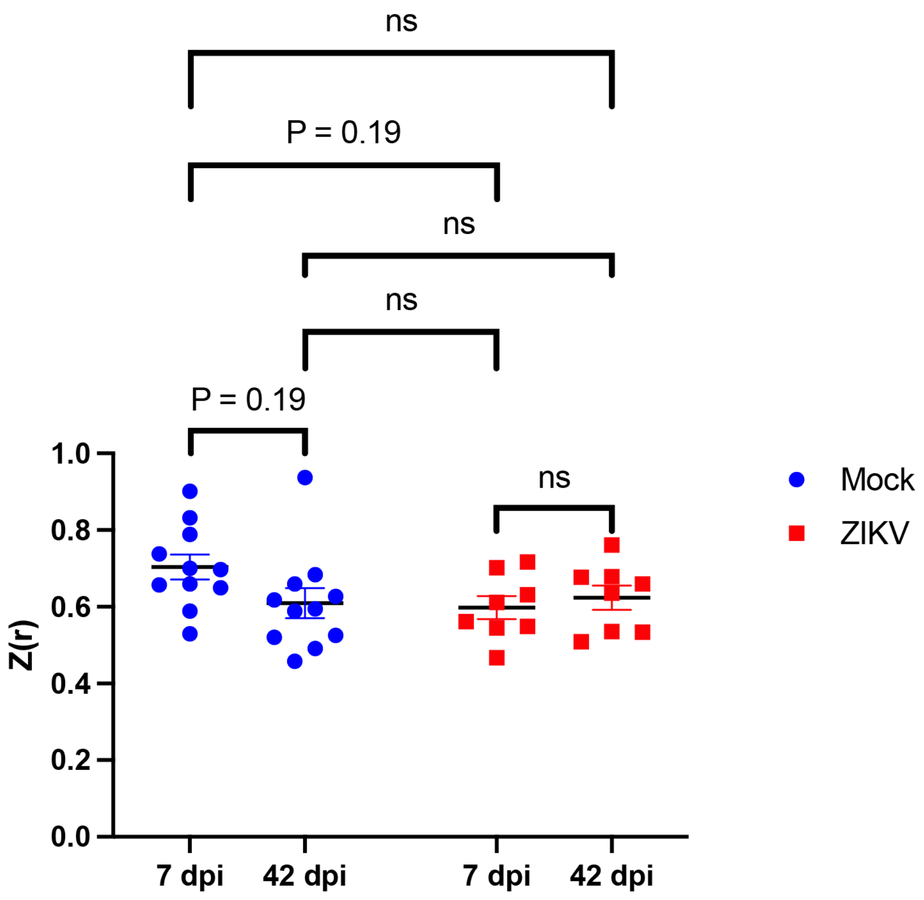

Figure S13. FC decreases with age in healthy mice. FC for all mock and ZIKV-infected mice at 7 dpi and 42 dpi, illustrating that even in mock mice, FC decreases between 7 dpi and 42 dpi. Statistical comparison by two-way ANOVA with correction for multiple comparisons.
